# On the role of sparseness in the evolution of modularity in gene regulatory networks

**DOI:** 10.1371/journal.pcbi.1006172

**Published:** 2018-05-18

**Authors:** Carlos Espinosa-Soto

**Affiliations:** Instituto de Física, Universidad Autónoma de San Luis Potosí, Manuel Nava 6, Zona Universitaria, San Luis Potosí, Mexico; Temple University, UNITED STATES

## Abstract

Modularity is a widespread property in biological systems. It implies that interactions occur mainly within groups of system elements. A modular arrangement facilitates adjustment of one module without perturbing the rest of the system. Therefore, modularity of developmental mechanisms is a major factor for evolvability, the potential to produce beneficial variation from random genetic change. Understanding how modularity evolves in gene regulatory networks, that create the distinct gene activity patterns that characterize different parts of an organism, is key to developmental and evolutionary biology. One hypothesis for the evolution of modules suggests that interactions between some sets of genes become maladaptive when selection favours additional gene activity patterns. The removal of such interactions by selection would result in the formation of modules. A second hypothesis suggests that modularity evolves in response to sparseness, the scarcity of interactions within a system. Here I simulate the evolution of gene regulatory networks and analyse diverse experimentally sustained networks to study the relationship between sparseness and modularity. My results suggest that sparseness alone is neither sufficient nor necessary to explain modularity in gene regulatory networks. However, sparseness amplifies the effects of forms of selection that, like selection for additional gene activity patterns, already produce an increase in modularity. That evolution of new gene activity patterns is frequent across evolution also supports that it is a major factor in the evolution of modularity. That sparseness is widespread across gene regulatory networks indicates that it may have facilitated the evolution of modules in a wide variety of cases.

## Author summary

Modular systems have performance and design advantages over non-modular systems. Thus, modularity is very important for the development of a wide range of new technological or clinical applications. Moreover, modularity is paramount to evolutionary biology since it allows adjusting one organismal function without disturbing other previously evolved functions. But how does modularity itself evolve? Here I analyse the structure of regulatory networks and follow simulations of network evolution to study two hypotheses for the origin of modules in gene regulatory networks. The first hypothesis considers that sparseness, a low number of interactions among the network genes, could be responsible for the evolution of modular networks. The second, that modules evolve when selection favours the production of additional gene activity patterns. I found that sparseness alone is neither sufficient nor necessary to explain modularity in gene regulatory networks. However, it enhances the effects of selection for multiple gene activity patterns. While selection for multiple patterns may be decisive in the evolution of modularity, that sparseness is widespread across gene regulatory networks suggests that its contributions should not be neglected.

## Introduction

Many biological systems are modular. That is, their interactions occur predominantly within groups of elements and rarely between groups [1–3]. Modularity seems to be associated to important attributes of distinct biological systems. For example, theoretical and experimental efforts have associated a modular organization to structural robustness in RNA [4] and proteins [5] and to the resilience of metapopulations [6]. The relevance of modularity extends beyond the scope of fundamental research in biology. The reason is that modularity confers design and functional benefits to distinct classes of systems. Therefore, for disciplines as diverse as evolutionary robotics [7, 8], artificial intelligence [9, 10], neuroscience [11, 12] and synthetic biology [13, 14], it is relevant to study the effects of a modular organization and how to construct modular systems. Advances in this direction may lead to new useful therapeutical and technological applications. Here, however, I focus on the role that modularity has in development and evolution.

Several researchers have underscored modularity in developmental mechanisms as an important property that facilitates evolution [1, 15–22]. The main underlying reason is that, in modular systems, perturbations of an element are often contained within its module and have few, little or no effects on the rest of the system [23]. It is thus possible to optimize a module without disturbing the functions of other modules. By allowing independent modification of different traits or functions, modularity increases the range of phenotypes that random genetic change can access. For example, the existence of distinct separate gene sets, i.e. modules, for beak width and length may have been an important factor in the evolution of a wide range of beak shapes in the Darwin finches’ adaptive radiation [24, 25]. That the production of distinct aspects of the colour pattern in different parts of butterfly wing blades depends on small and unique sets of genes has also indicated that modularity plays an important role in the evolution of a wide diversity of wing patterns in *Heliconius* butterflies [26].

Gene regulatory networks play fundamental roles in guiding developmental processes [27, 28]. They consist of sets of genes that cross-regulate their expression through regulatory interactions. Such interactions depend mainly on the production of transcription factors that bind cis-regulatory regions in other genes [28]. Upstream factors, be them genes external to the network, molecular signals coming from neighboring cells, environmental cues, or maternal factors, define a network’s initial state, in which some genes may be active and other genes may be inactive. Then, the genetically encoded interactions guide a dynamic process in which some genes change their activity state. Network dynamics eventually settles in a developmental end state, a gene expression pattern that indicates the network’s commitment to a particular task [29]. I will refer to such a terminal expression pattern as the network’s *gene activity phenotype* (GAP). The same gene network may yield different GAPs when subject to different signals that produce distinct initial states of gene activity. Thus, gene networks produce the different GAPs that distinguish tissues, organs and cell types. Evolution of new GAPs, through modification of gene regulatory networks, has produced many evolutionary innovations across the tree of life [30–32].

Given the relationship between modularity and evolvability, it is paramount to evolutionary biology to understand the origins of modularity. Because modularity refers only to how mechanisms are structured, and not directly to the phenotypic output of such mechanisms, modularity does not increase fitness by itself. Therefore, to understand how modules evolve we need to study how a modular arrangement relates to other properties [2]. Different groups have proposed distinct mechanisms to explain the evolutionary origins of modularity [2, 33–35].

Modularity may arise whenever interactions between distinct sets of genes obstruct adaptation [36]. In that case, selection would disfavour organisms with such deleterious interactions thus decreasing the number of interactions *between* sets. There are several hypotheses for what makes interactions between different sets of genes consistently deleterious. G.P. Wagner and collaborators suggested that interactions between genes associated to two different characters may be detrimental when one of such characters is subject to stabilizing selection and the other to directional selection. In this case, interactions between these groups of genes would obstruct the action of either form of selection [1, 17, 37]. In a different scenario, network modularity evolves when selection fluctuates recurrently between two selection regimes. In one regime, selection favours the performance of one complex task that combines two subfunctions. In the second regime, selection favours a different task that combines the same subfunctions in a different manner [38]. Hence, phenotypic optima vary in a modular manner. In this scenario, excessive interactions between elements involved in distinct subfunctions are deleterious. The reason is that networks that have different sets of elements assigned to different subfunctions and that combine subfunctions through few interactions, require less mutations to alternate from producing one optimum to producing the other. Thus, such modular networks have an evolutionary advantage. Congruently with this hypothesis, bacteria living in environments where fluctuations are more frequent tend to have metabolic networks with higher modularity scores [39].

Evolution of new gene activity phenotypes concerns another scenario where interactions between distinct sets of genes are selected against [40]. Specifically, modularity may increase if selection favours networks that produce an additional GAP, besides the network’s ancestral GAP. This scenario requires two sets of genes: one in which each gene has the same activity state in the ancestral and new GAPs (set A), and one in which each gene is inactive in one of the GAPs but active in the other (set B). Here, interactions between genes in the two sets obstruct adaptation: A gene in set A regulated mainly by genes in set B would likely present different activity states in the two GAPs. Alternatively, a gene in set B with a strong influence from set A would tend to show the same activity state in the new and ancestral GAPs. In other words, a gene that is more heavily influenced by genes in the other set than by genes in the same set would not comply with selection. In support of this scenario comes the observation that evolution of new gene activity phenotypes is frequent in an evolutionary scale. Such new GAPs are associated, for example, to the evolution of new cell types or to the specialization of serial homologues [30]. It is noteworthy that sister cell types [41–43] or specialized serial homologues [30, 44] tend to share the activity state of some genes but differ in that of others, thus conforming to this scenario’s requirements. Moreover, modularity is not lost once new GAPs evolve [40]. This contrasts with the ‘modular fluctuations’ scenario, in which modularity drops when fluctuations between selection regimes stop [38].

An alternative influential hypothesis for the origins of modules does not consider that interactions between specific sets of genes may be detrimental. Rather, it considers that *any* interaction is slightly deleterious and that network sparseness underlies modularity [45]. Clune and collaborators studied the evolution of feed-forward boolean networks as a model for the evolution of biological networks. They found that when connections come at a fitness cost and connectivity decreases, modular networks evolve. Importantly, many biological regulatory networks are sparse [46]. Moreover, later contributions suggested that sparseness is also associated to a hierarchic organization [47] and, in artificial neural networks, to an enhanced ability to learn new tasks [9].

How modularity is assessed is specially germane to discussions regarding the sparseness hypothesis for the origin of modules. Given a network structure, assume that there is an *a priori* proposal for a network partition *P* that assigns nodes to non-overlapping sets that serve as presumptive modules. The score *Q*_*P*_ reflects how interactions are concentrated within the modules that *P* assumes [3] (see Methods). However, when studying a network structure, one often has no preconception regarding the number or composition of modules. A common strategy is then to use an algorithm to search for the network’s *best* partition into modules [3, 48, 49]. That is, these algorithms attempt to find a partition that maximizes the modularity score. I will call *Q*^*opt*^ to the optimized modularity score that results from such a search. Because of local fluctuations in connection density, even networks that allocate their interactions in a random fashion, without any bias, may have random islands of nodes with connection densities greater than in the rest of the network [50–53]. It is noteworthy that random islands of a high connection density appear more easily in sparse networks [50, 51, 54]. For example, Guimerà et al found that *Q*^*opt*^ decreases with the number of connections in random Erdös-Rényi networks [50]. Thus, sparse random networks seem more modular than denser networks. These observations prompt the question of what is exactly the role of sparseness in the evolution of modularity in developmental gene regulatory networks. Is it only that sparse random networks have wider fluctuations in connection density or are there other effects? The question is specially relevant, considering the importance of modularity for development and evolution and the ubiquity of sparseness in biological networks.

To dissect the role of sparseness in the evolution of modularity, it may be useful to separate the effects that sparseness alone has on modularity from effects caused by other factors. One may do so by comparing a network’s raw modularity scores, *Q*^*opt*^ or *Q*_*P*_, with those of networks devoid of constraints other than preserving the number of connections and degree distribution. To attain such a comparison, I will use normalized scores $Q_{P}^{N}$ and *Q*^*N*^, that equal 0 when a network’s raw modularity score equals that expected for random networks with the same number of interactions (see Methods). A positive normalized score means that a network is more modular than networks with the same number of interactions.

**Fig 1:**
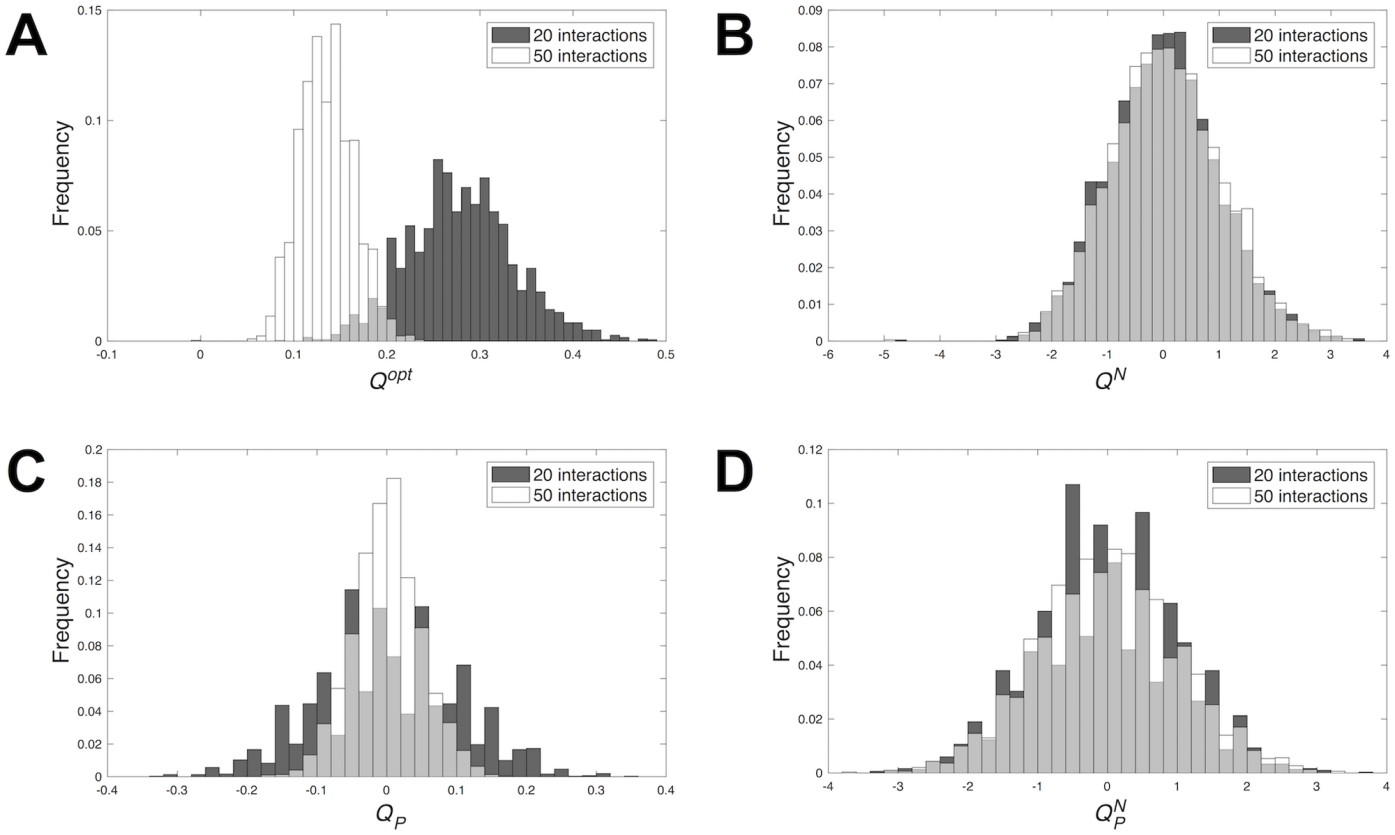
Distribution of modularity in random networks with different number of interactions. Each panel shows the distribution of a modularity score for sparse (20 interactions) and dense (50 interactions) networks with ten nodes. Each sample contains 3,000 random networks. (A) Distribution of the raw modularity score *Q*^*opt*^. Mean ± SD *Q*^*opt*^ equals 0.278 ± 0.057 for sparse and 0.135 ± 0.029 for dense networks. Kolmogorov-Smirnov test: *D* = 0.922; *p* < 2.2 × 10^−16^. (B) Distribution of the normalized modularity score *Q*^*N*^. Mean ± SD *Q*^*N*^ equals 0.008 ± 0.981 for sparse and 0.052 ± 0.991 for dense networks. Kolmogorov-Smirnov test: *D* = 0.064; *p* = 0.257. (C) Distribution of the raw modularity score *Q*_*P*_ for a specific partition *P*. Mean ± SD *Q*_*P*_ equals −0.002 ± 0.101 for sparse and 3.7 × 10^−4^ ± 0.05 for dense networks. Kolmogorov-Smirnov test: *D* = 0.22; *p* = 6.18 × 10^−11^. (D) Distribution of the normalized modularity score $Q_{P}^{N}$ for a specific partition *P*. Mean ± SD $Q_{P}^{N}$ equals −0.023 ± 1.02 for sparse and 0.007 ± 0.999 for dense networks. Kolmogorov-Smirnov test: *D* = 0.062; *p* = 0.29.

As a means of illustration, Fig 1 shows a comparison for two sets of random networks that differ in the number of interactions. Fig 1 first approaches modularity in these networks without any preconception of how they are partitioned into modules. Thus, modularity is assessed in Fig 1A using an algorithm that identifies partitions that optimize modularity [55]. This panel shows that, as indicated by the raw optimized modularity score *Q*^*opt*^, sparse networks seem to be more modular than networks with more interactions. Fig 1 also presents an analysis of the same sets of networks, but using the normalized modularity score *Q*^*N*^, that compares the raw score *Q*^*opt*^ to the expected value of the same score in networks with the same connectivity attributes. Fig 1B shows that the distribution of *Q*^*N*^ is practically the same for random networks with contrasting connectivities.

Fig 1C shows a comparison of the same sets of networks, but now in terms of *Q*_*P*_, the raw modularity score associated to a specific partition *P* that, for this example, I chose arbitrarily. The distribution of *Q*_*P*_ is centered at 0 for both sparse and dense networks. However, Fig 1C also shows that sparse and dense networks differ in how easily a random network’s *Q*_*P*_ score tends to deviate from 0. Notwithstanding, Fig 1D shows that, if we evaluate the modularity associated to partition *P* using the normalized score $Q_{P}^{N}$, random networks with contrasting connectivities present very similar means and spread. In sum, normalized modularity scores may be a better indicator of the deviation in modularity with respect to random expectation, regardless the number of connections. They allow to discard the effect that sparseness alone has on modularity.

One may distinguish three non-exclusive possible manners in which sparseness could contribute to modularity in developmental gene regulatory networks: i) Sparseness may suffice to explain modularity in such networks. If this were the case, gene regulatory networks would be as modular as random networks with the same number of interactions. ii) Sparseness may be necessary for the evolution and consolidation of modules. In this case, evolution of modularity would be impossible in dense networks. iii) Sparseness may enhance the effects of other mechanisms. To assess the role of sparseness in the evolution of modularity, I designed computer simulations of the evolution and dynamics of gene networks and analyses of the structure of biological regulatory networks. I report that sparseness is neither sufficient nor necessary to explain modularity in gene regulatory networks. Notwithstanding, I also found that sparseness has a positive effect on the evolution of modularity when it is combined with additional mechanisms. In sum, this study suggests that, despite its positive contribution, sparseness is not a critical factor for the evolution of modularity. Moreover, the analyses that I put forward are consistent with the proposal that modularity evolves easily under selection for new additional gene activity phenotypes, where interactions between distinct sets of genes are deleterious. That gene regulatory networks are indeed sparse facilitates the evolution of a modular arrangement.

## Methods

### Model of developmental process: Network dynamics

The work that I present here considers both the development and evolution of gene regulatory networks. In terms of gene networks, development entails the production of stable GAPs through regulatory interactions among a network’s genes.

To model a gene regulatory network’s developmental dynamics, I consider a set of *N* nodes that represent cross-regulating genes. A vector **s**^*t*^ describes the system state at a given time *t* by listing the activity state of each gene at that moment. At time *t*, a gene *i* may be active ($s_{i}^{t}=1$) or inactive ($s_{i}^{t}=0$).

**Fig 2:**
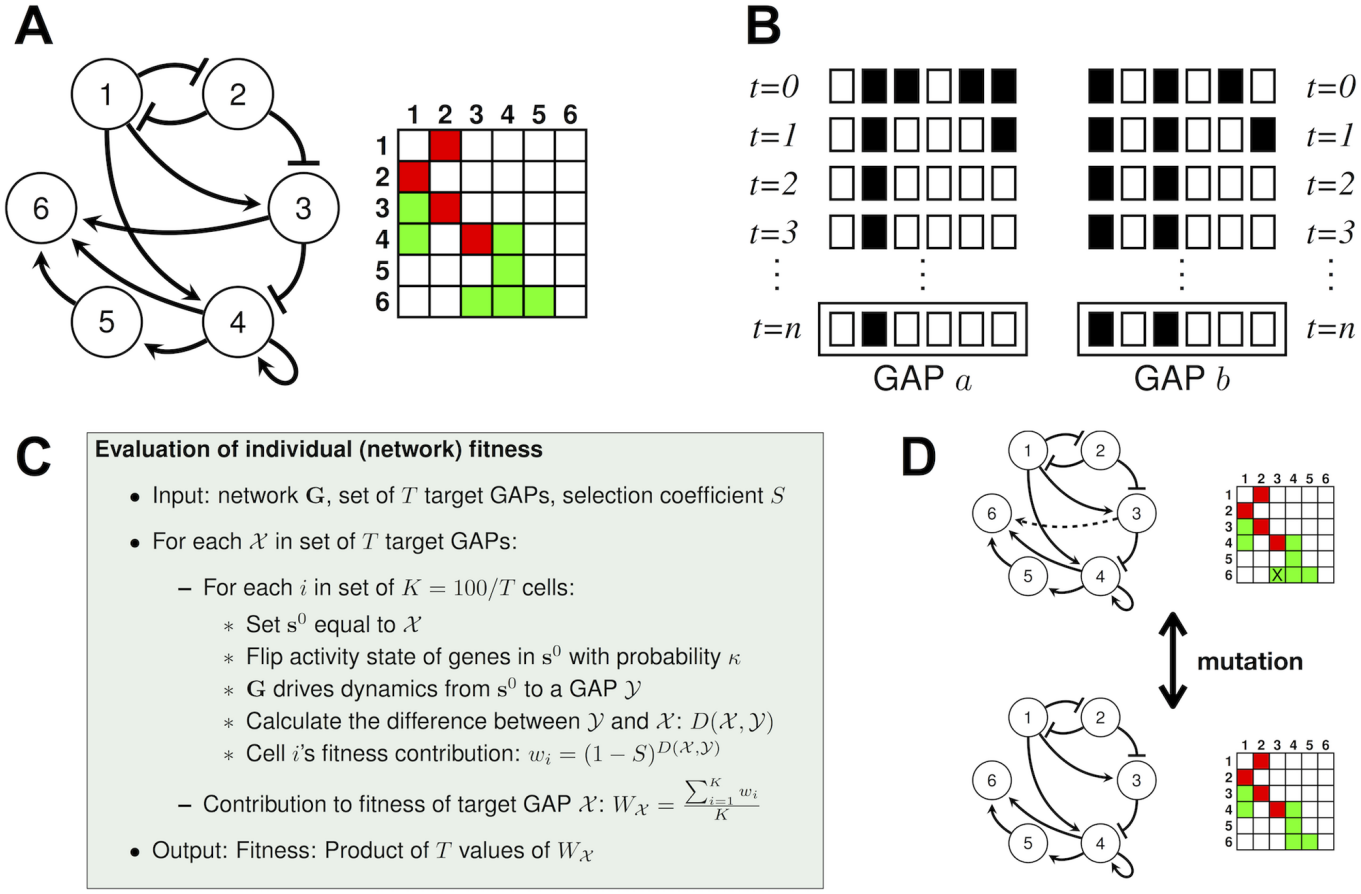
Gene network dynamics, mutation and evaluation of fitness. (A) A gene network can be described as a graph or as a matrix in which positive entries (green squares) represent activatory interactions (arrows) and negative entries (red squares) represent inhibitory interactions (blunt-end lines). (B) Each row with six squares represent a system state. White and black squares represent active and inactive genes, respectively. An initial pattern of gene activity (at *t* = 0) is updated according to the interactions described in panel (A) until dynamics lead to a GAP, a sequence of system states that is repeated indefinitely. The gene network in (A) is able to yield two different GAPs when it starts its dynamics from different initial system states. (C) Summary of the procedure to evaluate a network’s fitness in evolutionary simulations. Fitness is higher in networks that produce GAPs, from different initial conditions, similar to target GAPs that selection favours. (D) Mutations in the model change interactions between genes. They thus modify the matrix that specifies whether a gene *i* regulates a second gene *j*. The activatory interaction that mutation wrecks is dashed in the top network and its corresponding entry is marked with an X.

In nature, an organism’s genome defines interactions between regulatory genes, mainly through specification of cis-regulatory regions that transcription factors bind [28]. In the model, who regulates whom is defined in a matrix **G** that represents an organism’s genotype (Fig 2A). A positive entry in matrix **G**, *g*_*ij*_ > 0, indicates that gene *j* favours the expression of gene *i*. In contrast, a negative entry *g*_*ij*_ < 0 means that gene *j* inhibits the activity of gene *i*. The change in gene activity depends on:

$$\begin{array}{c} s_{i}^{t+1}=\sigma_{i}[\sum_{j=1}^{N}g_{ij}s_{j}^{t}-\theta_{i}] \end{array}$$ (1)

where *σ*_*i*_ is a step function given by

$$\begin{array}{c} \sigma_{i}\left(x\right)=\{\begin{array}{cc} 1\text{,} & \text{if}\;x>0\\ s_{i}^{t}\text{,} & \text{if}\;x=0\\ 0\text{,} & \text{if}\;x<0 \end{array} \end{array}$$ (2)

Thus, a gene *i* becomes active if the sum of the influence of *i*’s *active* regulators surpasses a threshold *θ*_*i*_ specific for gene *i*. I consider that the value of *θ*_*i*_ depends on gene *i*’s regulators. Specifically, $\theta_{i}=\frac{\sum_{j=1}^{N}g_{ij}}{2}$. This value of *θ*_*i*_ guarantees the existence of combinations of activity states of *i*’s regulators that can switch gene *i* on or off. Moreover, with this definition of *θ*_*i*_ and *σ*_*i*_, the model is equivalent to a model of gene network dynamics first used by Wagner to study evolutionary properties of gene regulatory networks [56, 57]. Variations of this model have allowed researchers to address successfully several diverse important questions on the evolution of regulatory networks [58]. Importantly, previous research [59] has shown that this model has properties that allow the generalization of results that one may find for one GAP to other easily recognizable GAPs (see details in section 1.1 of S1 Text).

In nature, a network’s dynamic trajectory describes the changes in gene activity that a cell experiences until gene activity settles. The network has then reached a developmental end state, that is, a GAP. Network dynamics starts from an initial state, defined by factors external to the network. In the model, the system starts its dynamics from an initial system state **s**^0^ and network genes update their activity state iteratively according to Eq 1. Eventually, because the system is discrete, it attains a state that it has visited previously. In the absence of perturbations, the system then follows the same dynamic trajectory and thus becomes locked in a sequence of *k* distinct system states that represents a GAP (Fig 2B). The network has then reached a developmental end-state [60]. If *k* = 1, the GAP is stationary. In it, a gene activity pattern self-maintains (Fig 2B). If *k* > 1, the GAP is a limit cycle in which a particular system state reappears every *k* iterations. The same network genotype may attain distinct GAPs when it starts its dynamics from different initial conditions (Fig 2B). In nature, such different initial conditions may arise in distinct parts of an organism subject to different molecular signals.

### Evaluation of a network’s fitness

To assess an individual’s phenotype and evaluate its fitness (Fig 2C), I consider the network’s ability to produce, from different initial conditions, *T* distinct reference GAPs that serve as evolutionary goals. I will refer to them as target GAPs. I consider that each individual is composed of 100 cells and assigns *K* = 100/*T* of them to each of the *T* functions that the distinct target GAPs optimally perform. A cell required to produce target GAP X starts its dynamics from an initial condition that I obtain by flipping with probability *κ* each entry in the target GAP X. *κ* thus reflects how often the sequences of changes in gene activity are perturbed by changes in the initial system state. In nature, such perturbations may arise from developmental noise, for example in the form of stochastic fluctuations in the number of protein molecules, or from random disturbances in a cell’s environment. A cell’s dynamic trajectory stops once the network attains a GAP Y, according to the procedure described in the preceding section. A cell’s contribution to fitness depends on how different Y is from the target GAP X in the following manner:

$$\begin{array}{c} w={\left(1-S\right)}^{D(X,Y)} \end{array}$$ (3)

where *S* is the selection coefficient, that calibrates how deleterious are deviations from a target GAP. D(X,Y) measures how different are Y and X (see section 1.2 in S1 Text).

I define the contribution of a target GAP X to organismal fitness as the arithmetic mean of the fitness contribution of the *K* cells required to produce X. Organismal fitness is the product of the fitness contribution of each target GAP.

### Evolution of network populations

A population is first created as copies of a founder network. To create such a founder network, I built random networks with *γN*^2^ interactions until one network appears that produces, in the absence of perturbations of the initial system state, the target GAP that selection favours when evolution starts. Then, I subject network populations to rounds (*i.e*. generations) of selection and mutation to simulate an evolutionary process. To produce the next generation, I first evaluate the fitness of each network by assessing how similar are the GAPs that it yields to target GAPs, as explained in the previous section. Then, I pick randomly (with replacement) *M* networks with roulette wheel selection, in which the probability to pick a network is proportional to its fitness. Each gene in a network is then subject to mutation with probability *μ*.

Mutation changes an entry in the matrix **G** that lists gene interactions. It thus changes the number of regulators that a gene has (Fig 2D). Gene duplication and other kinds of mutations are not considered in this contribution. As described in detail in S1 Text (section 1.3), the propensity to gain interactions, *γ*, modulates how likely it is that a new mutation leads to a new interaction and not to the loss of an existing interaction. The probabilities that a gene *i* loses or acquires an interaction also depend on the number of regulators that *i* already has. Acquiring or losing an interaction is more likely when the number of *i*’s regulators is low or high, respectively. In this setup, mutation tends to pull the number of a gene’s regulators towards *Nγ*. Thus, tuning *γ* allows to define the expected number of interactions per network *γN*^2^.

The analyses that I present here concern those populations in which maximum fitness surpassed a threshold of 0.9. For each of such populations, I chose randomly one network among those with the highest fitness in that population. Notwithstanding, the results are qualitatively the same when including all populations, regardless whether they adapted successfully or not (S1 Fig), and when I consider average population values in successfully adapted populations (S2 Fig). Unless stated otherwise, the parameters that I use in the simulations presented here are: *N* = 10, *κ* = 0.05, *γ* = 0.3, *S* = 0.4, *μ* = 0.01 and *M* = 200. With this choice of parameter values, the optimal phenotype evolved in most cases, without incurring in excessive computational cost. Indeed, even in scenarios where adaptation is slower, evolution of modularity had effectively come to a halt by the end of the simulations (see section 2.1 in S1 Text). Many of the evolutionary scenarios that I address include two distinct stages. The procedures described above are followed in both stages, albeit perhaps with different target GAPs, or with a different value for a specific parameter that affects evolution. The first stage merely sets an ancestral population. Comparing populations before and after evolution under different conditions allows addressing if those conditions increase modularity relative to the ancestral state.

### Evaluation of modularity

Given a directed network and a specific partition *P* of the network’s nodes into non-overlapping sets, the network’s modularity, *i.e*. the degree with which interactions are concentrated within the partition sets, is described with:

$$\begin{array}{c} Q_{P}=\sum_{m}[\frac{l_{m}}{L}-\frac{d_{m}^{in}d_{m}^{out}}{L^{2}}] \end{array}$$ (4)

where *m* refers to one of the sets of nodes that compose partition *P*, *L* is the total number of regulatory interactions in the network, *l*_*m*_ is the number of interactions within module *m*, $d_{m}^{in}$ is the sum of incoming interactions of all nodes in module *m* and $d_{m}^{out}$ is the same but for outgoing interactions [55]. Because random networks that differ in the number of connections or in other networks properties vary in how easily they deviate from random expectation, I compare a network’s *Q*_*P*_ with that of networks with the same number of nodes, interactions and degree distribution but devoid of any other constraints. Hence, I measure the *Q*_*P*_ score for the same partition *P* in a set of 10^3^ networks that allocate their interactions randomly but that preserve the same degree distribution and the same number of nodes and edges as the original network. To create randomized networks I follow the ‘switching’ algorithm [61]. Thus, I assess a normalized version of the *Q*_*P*_ score that measures modularity after discarding the modularity due to sparseness alone:

$$\begin{array}{c} Q_{P}^{N}=\frac{Q_{P}-{\hat{Q}}_{P}}{SD_{Q,P}} \end{array}$$ (5)

where ${\hat{Q}}_{P}$ and *SD*_*Q*,*P*_ refer to the mean and standard deviation of *Q*_*P*_ for networks in the randomized set.

In the lack of an hypothesis for the composition of modules in the network, an usual strategy is to search for a partition that maximizes *Q*_*P*_ [3, 48, 49]. Here, I will call *Q*^*opt*^ to the modularity score that results from such an optimization procedure. In this paper, I use the spectral method proposed by Newman, known for its advantages in performance and computational cost [49]. Specifically, I use Leicht and Newman’s version of the method that allows its application to directed networks [55].

Because even random networks can have a high *Q*^*opt*^ value, it is convenient to compare a network’s *Q*^*opt*^ with the random expectation for networks with the same properties. For each network under study, I built a set of 10^3^ randomized networks with the same number of nodes, edges and degree distribution. I then apply Leicht and Newman’s spectral method to each network in the set. Next, I use a z-score as a normalized modularity score:

$$\begin{array}{c} Q^{N}=\frac{Q^{opt}-\hat{Q}}{SD_{Q}} \end{array}$$ (6)

where $\hat{Q}$ and *SD*_*Q*_ refer to the mean and standard deviation of *Q*^*opt*^ for networks in the random set. In order to enable comparisons with previous work, section 2.2 in S1 Text provides the main results in this article, but from the perspective of the raw optimized score *Q*^*opt*^.

## Results

### Sparseness alone does not explain modularity in gene regulatory networks

Previous research supports that network modularity evolves when networks become sparser [9, 45, 47, 62]. However, one must take into account how modularity is regularly assessed. In the absence of an hypothesis for which nodes are integrated into modules, a network’s modularity is usually evaluated by finding one network partition that maximizes the modularity score [48, 49, 63]. I refer to such an optimized score as *Q*^*opt*^. Because the search algorithms look for the *best* partition, even random networks can have a high *Q*^*opt*^ score [50–53]. Sets of nodes with a connectivity (locally) higher than in the whole network appear more easily in sparse than in densely connected random networks. As explained previously, normalized modularity scores allow fairer comparisons of a network’s modularity in terms of how it deviates with respect to random networks with the same number of connections. Therefore, hereafter I will report a network’s modularity using normalized scores. I will use score $Q_{P}^{N}$ whenever I consider a mechanism that predicts the composition of modules. In this case, $Q_{P}^{N}$ tells whether interactions concentrate specifically according to the proposed partition *P*. If different sets with high internal connectivity were to arise, they should not validate the mechanism under evaluation for its capacity to create and consolidate modules. When an hypothesis for the composition of modules is lacking, I will use the score *Q*^*N*^ that compares an optimized score in a focal network to similarly optimized scores in randomized networks.

One open possibility is that modularity in developmental regulatory networks is not higher than expected by chance for networks of the same size and connectivity. If that were the case, what we perceive as modules would be the result of local fluctuations in connection density that theory predicts in sparse random networks [50, 51, 54]. Then, the normalized modularity score of developmental networks would follow a distribution similar to that in Fig 1B, with (nearly) equal probability of being positive as that of being negative. To assess this possibility, I revisited 12 recent studies of developmental regulatory networks. Out of the many possible sources of information, I took the network structures from modelling studies sustained on experimental evidence. The reason is that the confidence is high that a model that successfully reproduces a developmental process takes into account all critical factors and interactions involved in the process. Each of these networks can attain any of several stable gene activity phenotypes (GAPs), as they participate in developmental decisions in plants and animals (Table 1). The sample contains studies in plants [64–66], insects [67], nematodes [68], echinoderms [69] and mammals [70–75]. The networks vary greatly in size (ranging from 5 to 94 nodes) and number of interactions (13-209). Connection density does not span a wide interval (1.59 to 3.24 interactions per node), coinciding with the observation that regulatory networks are usually sparse [46]. I found that all twelve networks have a positive normalized modularity score *Q*^*N*^ (Table 1). In other words, the modularity of each network is higher than the average for random networks of the same size, number of interactions and degree distribution. Moreover, in most cases *Q*^*N*^ is not close to 0, which is the null expectation. Had *Q*^*N*^ in these developmental networks come from a symmetric distribution centered on 0, the probability that all twelve scores were positive would be 0.5^12^ ≈ 2.4 × 10^−4^. These observations support that networks in development tend to be more modular than expected for random networks with the same connectivity. Hence, sparseness alone is unlikely to explain modularity in gene regulatory networks. Moreover, that developmental networks that produce multiple GAPs have a positive normalized modularity score is consistent with the proposal that evolution of new additional gene activity phenotypes may be an important factor for the evolution of modularity [40].

**Table 1:** Modularity in developmental multistable regulatory networks.

System	Nodes	Interactions	*Q*	$\hat{Q}$	*SD*_*Q*_	*Q*^*N*^
Arabidopsis floral organ determination [64]	15	43	0.318	0.223	0.033	2.895
Sea urchin endomesoderm development^a^ [69]	79	209	0.531	0.358	0.015	11.257
Terminal differentiation of B cells [75]	22	39	0.381	0.366	0.038	0.389
Macrophage activation^b^ [74]	94	170	0.59	0.482	0.017	6.445
Primary sex determination in eutherians^c^ [72]	18	40	0.372	0.292	0.035	2.253
Specification of mouse ventral neural tube^d^ [70]	17	33	0.403	0.339	0.039	1.639
Pancreas development [73]	5	13	0.26	0.154	0.065	1.629
Drosophila mesoderm specification^c^ [67]	48	78	0.498	0.462	0.024	1.533
Lymphoid and myeloid cell specification^e^ [71]	21	68	0.244	0.218	0.024	1.097
Vascular bundle differentiation [65]	22	35	0.491	0.431	0.04	1.476
Arabidopsis root stem cell niche [66]	11	34	0.384	0.205	0.039	4.586
Differentiation in the *C. elegans* vulva [68]	14	38	0.37	0.262	0.043	2.535

^a^: The network’s “*view from the genome*” was downloaded on October 27, 2017 (http://grns.biotapestry.org/SpEndomes/). Maternal, ubiquituous, unknown unregulated and isolated factors were omitted.

^b^: Protein and mRNA nodes corresponding to the same gene were fused into a single node. Housekeeping factors were omitted.

^c^: A reduced version of the network produces, qualitatively, the same results.

^d^: The network’s “*view from the genome*” was downloaded on October 30, 2017 (http://grns.biotapestry.org/VNT/).

^e^: Activated and unactivated versions of two receptors were fused into a single node.

### Selection for additional GAPs and the evolution of modularity

I next used a model of gene network dynamics (Fig 2) to study how modularity may evolve. The model allows to follow the changes in gene activity according to the regulatory interactions that the network specifies. Eventually network dynamics reaches a GAP, a pattern of gene activity in which the system settles. Importantly, a network may produce different GAPs, for example, when it starts its dynamics from different initial system states that may occur in different parts of an organism (Fig 2B). Because gene interactions guide developmental dynamics, the network structure defines the GAPs that a network is able to produce. Therefore, changes in gene interactions may produce changes in the GAPs that a network attains. I subjected network populations to cycles of mutation and selection. On the one hand, a mutation changes an interaction at random (Fig 2D). On the other hand, selection favours those networks that were able to produce gene activity phenotypes similar to predefined target GAPs that are assumed to perform a biological function optimally. Thus, the networks that had greater chances of leaving offspring for the next generation were those that produced the target GAPs even under perturbations of the network dynamics (see Fig 2C and Methods).

**Fig 3:**
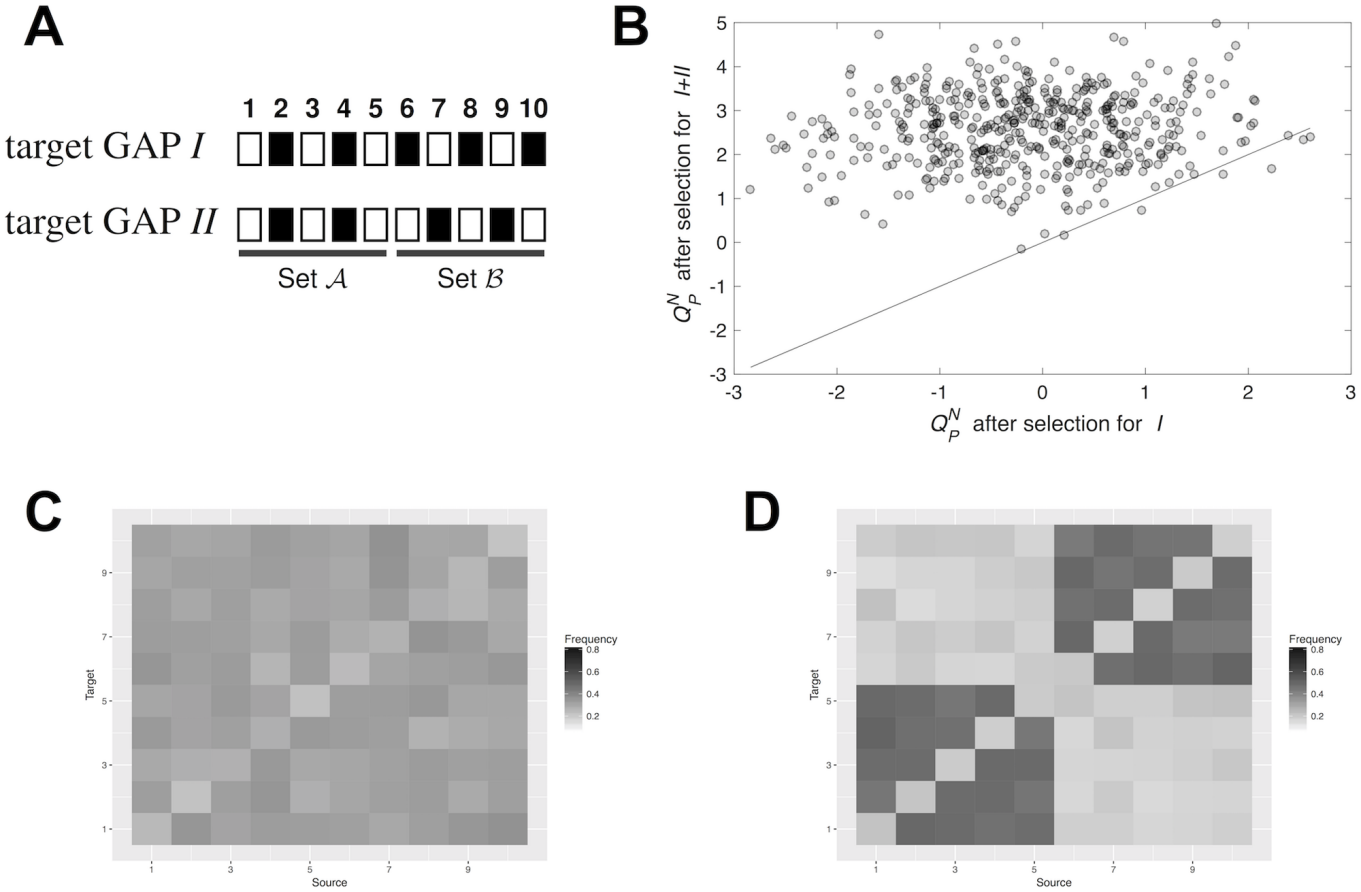
Modularity evolves after selection for an additional gene activity phenotype. (A) Target GAPs *I* and *II*. White and black squares represent active and inactive genes, respectively. Genes 1-5 are grouped in set A and genes 6-10 are grouped in set B. Note that genes in set A have the same activity state in both target GAPs. In contrast, genes in set B have a different activity state in both target GAPs. Networks evolve in a first stage under selection to produce target GAP *I*. In a second stage, selection favours networks that produce target GAPs *I* and *II* from distinct initial system states, that may occur, for example, in cells in different parts of an organism. (B) $Q_{P}^{N}$ increases after selection for an additional gene activity phenotype. Each dot represents an independently evolving population. The horizontal axis indicates the $Q_{P}^{N}$ score for a network with the highest fitness before starting selection for the two GAPs. The vertical axis denotes the same score but for a network after selection for both GAPs. The solid diagonal is the identity line. (C) After selection for GAP *I* alone, interactions between any pair of genes occur with similar frequency. Grayscale indicates the fraction of independently evolved networks that have a certain source-target regulatory interaction. (D) After selection for both target GAPs *I* and *II* interactions occur mainly either between genes in set A or between genes in set B.

Simulations of the evolution of gene regulatory networks have already suggested that networks that evolve to produce both a new and an ancestral GAPs from different initial conditions tend to be more modular than ancestral networks [40]. As explained in the introduction, the reason would be that interactions between distinct sets of genes obstruct the production of either the old or the new GAP. Specifically, previous research had shown that evolution of new GAPs increases *Q*^*N*^ [40]. Hence, I first assessed whether the modularity score $Q_{P}^{N}$ associated to a partition *P* also increases in this scenario. To address this question, I considered an ancestral target GAP *I*, a new additional beneficial target GAP *II* and two sets of genes A and B of equal size. Each gene in set A has the same activity state in target GAPs *I* and *II*. In contrast, each gene in set B has a different activity state in target GAPs *I* and *II* (Fig 3A). Thus, the specific partition that I consider, *P*, allocates genes in set A to one module and genes in set B to a second module, as previous research predicts [40]. Then, I followed the evolution of 500 populations, each with *M* = 200 individuals (i.e. networks), for 2,000 generations of selection for target GAP *I*. This first stage of evolution sets ancestral populations before the evolution of a new gene activity pattern. In a second stage of evolution, lasting 8,000 generations, selection favours those individuals that yield GAP *I* in half of their cells and that produce, from different initial conditions, GAP *II* in the remaining cells. At the end of each selection regime, I measured $Q_{P}^{N}$ in a network with the highest fitness in each population. 475 populations successfully adapted to both selection regimes. The mean $Q_{P}^{N}$ value for networks after selection for *I* is -0.14 (SD = 1.009). After selection for both target GAPs, mean $Q_{P}^{N}$ equals 2.569 (SD = 0.846). The difference is highly significant according to a Wilcoxon signed-rank test (Fig 3B; *W* = 112, 990; *p* < 2.2 × 10^−16^). The consolidation of two distinct modules, each comprising genes in either set A or B, is also evident by looking at how interactions are distributed at the end of each selection regime. Fig 3C shows that when selection favours target GAP *I* alone interactions between any pair of genes appear with similar frequency. In contrast, after selection for both target GAPs *I* and *II*, interactions are concentrated within sets A or B (Fig 3D). These observations are consistent with the hypothesis that selection for additional GAPs increases modularity.

### The effects of sparseness on the evolution of modularity

**Fig 4:**
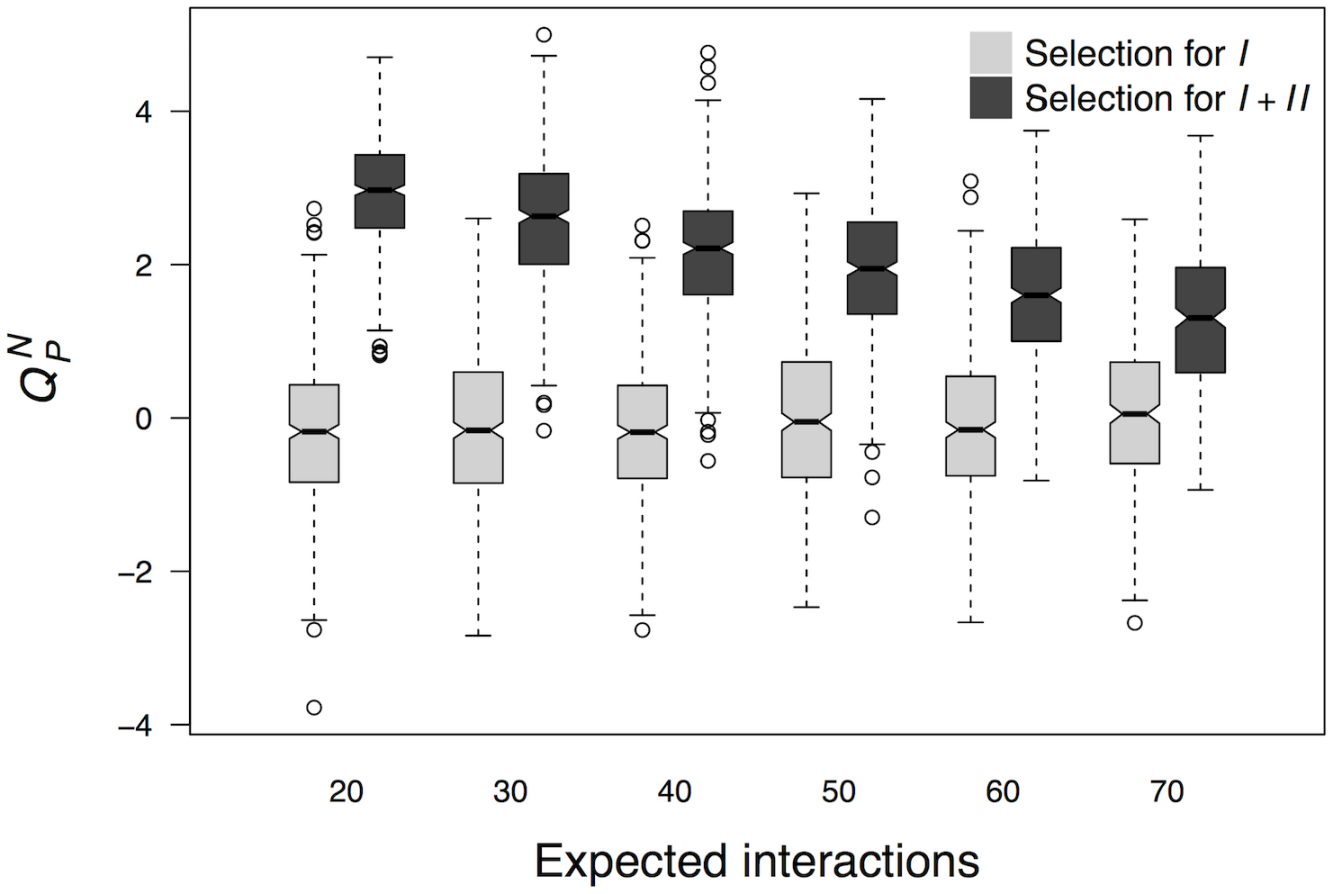
Selection for two GAPs produces a greater increase in modularity in sparser networks. The selection regimes are the same as in Fig 3. In a first stage, selection favoured networks that produced target GAP *I*. In a second stage, networks with highest fitness were those that produced GAPs *I* and *II* in cells with different initial system states (Fig 3A). The $Q_{P}^{N}$ score refers to a partition *P* that assigns the first five genes to one set and the rest of the genes to another. The number of expected interactions is modulated through *γ*, the relative propensity to acquire interactions.

Sparseness may still play a role in the evolution of modularity, even if it is not sufficient to increase modularity to the extent observed in gene regulatory networks. Thus, I addressed whether sparseness has a role in increasing modularity beyond the level expected in random networks. To pursue the answer, I evolved 3,000 populations of networks, 500 with each of six different values for the propensity to gain interactions *γ*. As explained in Methods, the expected number of interactions in evolving networks increases linearly with increasing *γ*. Network populations evolved in the same scenario as populations in Fig 3: 2, 000 generations under selection for target GAP *I* and then 8, 000 generations under selection for target GAPs *I* and *II* (Fig 3A). These simulations show that a greater number of interactions makes adaptation more difficult in this evolutionary scenario, as the number of populations that successfully adapted decreased with *γ*. The number of populations in which networks surpassed a fitness threshold of 0.9 at the end of the two selection regimes was 485, 475, 459, 441, 391 and 293 when *γ* equaled 0.2, 0.3, 0.4, 0.5, 0.6 and 0.7, respectively. I assessed the modularity score $Q_{P}^{N}$, referring to a partition *P* into sets A and B, in networks with the highest fitness in populations that adapted successfully. The increase in modularity is less pronounced when interactions accumulate more easily (Fig 4). Notwithstanding, there is a substantial increment in modularity even in networks with a propensity to acquire interactions as unrealistically high as 0.7. In this case, mean $Q_{P}^{N}$ equals 0.065 (SD = 1.013) after selection for target GAP *I* and it equals 1.292 (SD = 0.964) after selection for *I* and *II*. This increase in modularity is also statistically significant (*W* = 38, 418; *p* < 2.2 × 10^−16^).

Even though sparseness is not *sufficient*, it may well be *necessary* to increase modularity above levels expected in random networks. If this were the case, the increase in modularity would only occur in networks with few connections, even in regimes where propensity to gain interactions is high. This possibility may seem supported by the observation that adaptation to produce two target GAPs and the increase in modularity are more likely when the probability to gain interactions is low. However, this is not the case. The expected number of interactions for networks evolving in the absence of selection is 20, 30, 40, 50, 60 or 70 when *γ* equals 0.2, 0.3, 0.4, 0.5, 0.6 or 0.7, respectively. Similarly, the average ± SD numbers of interactions in networks successfully adapted to produce target GAPs *I* and *II* are, respectively, 21.38 ± 3.62, 30.1 ± 4.34, 39.69 ± 4.67, 50.25 ± 4.97, 60.78 ± 4.79 and 70.63 ± 4.54. One may think that the number of interactions is so close to the number of expected interactions only because mutation drives too strongly the number of interactions towards values close to *γN*^2^ (see Methods and section 1.3 in S1 Text). Nevertheless, even when mutation is not biased towards a specific number of regulators per gene, the number of interactions does not decrease beyond random expectation after selection for an additional GAP (S3 Fig). All these results indicate that networks do not tend to lose connections when evolving modularity under selection to produce two different target GAPs. Consequently, modularity can evolve in non-sparse networks. Moreover, although computational cost prohibits exploration of the evolution of very large networks, simulation of the evolution of slightly larger networks in the same evolutionary scenario are also consistent with these observations (S4 Fig).

It is noteworthy that the greater increase in modularity in networks with a lower propensity to acquire interactions is also observed after selection for *I* and *II* when the model of network dynamics is modified substantially. Specifically, this effect is observed when the gene activity threshold *θ*_*i*_ (Eq 1) is set to zero for all genes (S5 Fig) and when the entries in genotype matrices **G** are continuous (S6 Fig). These results suggest that the effect of sparseness on the evolution of modularity after selection of an additional target GAP does not depend on the model’s details.

For reasons explained in section 1.1 in S1 Text, what is true for networks that evolve to produce target GAPs *I* and *II* will also be valid if another pair of stationary GAPs had taken the place of *I* and *II* in the simulation. The only restriction is that the two GAPs should differ in the same number of activity states as *I* and *II*. Notwithstanding, I tested whether a low value of *γ* is associated to a greater increase in modularity when the pairs of target GAPs that selection favours present more and less differences in gene activity than *I* and *II*. S7 and S8 Figs show that this effect is also observed in simulations in which the two target GAPs differ in the activity of three and seven genes, respectively. Therefore, these observations are not particular to networks evolving GAPs *I* and *II*, but extend to networks evolving a wide variety of pairs of target GAPs.

The simulations that I have already presented suggest that sparseness contributes to the evolution of modularity, despite being neither necessary nor sufficient. An additional analysis supports this interpretation. I evolved 500 network populations under selection to produce target GAPs *I* and *II* for 2 × 10^4^ generations. In the course of the first 10^4^ generations, the propensity to gain interactions was relatively high (*γ* = 0.4). In the last 10^4^ generations *γ* equaled 0.2. Although $Q_{P}^{N}$ was already high after the first 10^4^ generations of evolution (mean: 2.17; SD: 0.874), it rises substantially after evolution with a low *γ* (mean: 2.885; SD: 0.75). This increment is also statistically highly significant (*W* = 83, 538; *p* < 2.2 × 10^−16^).

It is noteworthy that the positive effects that sparseness has on modularity do not occur under any selection regime. I studied the evolution of networks under selection for a single target GAP, specifically GAP *I*. As in the previous analyses, I considered two epochs, each lasting 10^4^ generations. The propensity to gain interactions, *γ*, equaled 0.4 and 0.2 in the first and second epoch, respectively. Because of the lack of an hypothesis for a partition in this selection regime, I assessed the score *Q*^*N*^ instead of $Q_{P}^{N}$. At the end of each of the two epochs modularity was lower than that of random networks with the same connectivity. Mean *Q*^*N*^ equaled -0.44 (SD = 0.98) when *γ* had a high value. After evolution under a low *γ*, mean *Q*^*N*^ equaled -0.63 (SD = 0.88). Indeed, a Wilcoxon signed-rank test does not support that modularity increased after decreasing the value of *γ* (*W* = 51, 492;*p* = 0.999).

These results together support that sparseness may enhance the positive effects that selection has on taking modularity further above values expected in random networks. Remarkably, these positive effects do not appear in any selection regime. They do appear when interactions between distinct sets of genes obstruct adaptation. Otherwise, sparseness does not seem to contribute to the appearance and consolidation of modules beyond random expectation.

### Enhancing effects on the evolution of modularity are not unique to sparseness

Are there any other factors that, like sparseness, modulate the increase in modularity that other mechanisms produce? I considered that non-genetic perturbations in a network’s initial system state may also play this role. That is, it may be that greater probabilities of altering the dynamic trajectory that leads to a GAP may contribute to increased modularity. The reason would be that the detrimental effects of some interactions between modules may appear only under some combinations of gene activity states but not in others. Thus, trying more and different trajectories may facilitate revealing these conditionally deleterious interactions. In nature, such perturbations in dynamic trajectories may come from developmental noise or from environmental disturbances. In the model, I simulate them by perturbing initial states in network dynamics with probability *κ* (see Methods).

**Fig 5:**
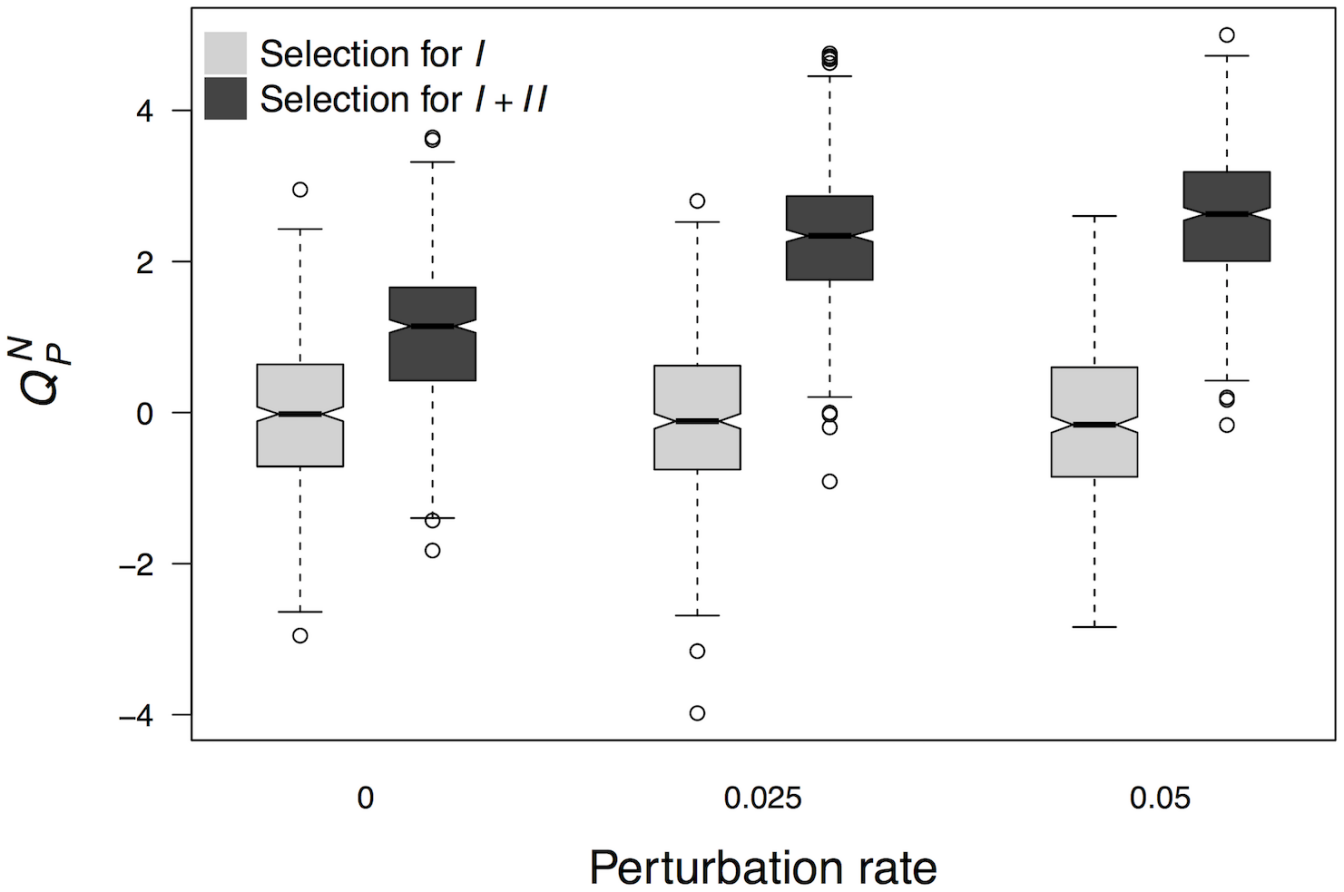
The rate *κ* of perturbation of initial system states contributes to the increase in modularity due to selection for two GAPs. The increase in modularity after selection for two target GAPs is greater when evolution occurs under higher rates of non-genetic perturbation. The selection regimes are the same as in Fig 3. In a first stage, selection favoured networks that produced target GAP *I*. In a second stage, networks with highest fitness were those that produced GAPs *I* and *II* from different initial system states (Fig 3A). $Q_{P}^{N}$ refers to a partition *P* that assigns the first five genes to one set and the rest of the genes to another.

I evolved network populations under different probabilities *κ* of perturbing initial system states. As in previous simulations, the first 2 × 10^3^ generations selection favoured networks that produced target GAP *I*, while the remaining 8 × 10^3^ generations selection benefitted networks yielding target GAPs *I* and *II*. Similarly as with sparseness, Fig 5 shows that as perturbation rate takes higher values, the increase in $Q_{P}^{N}$ is greater after selection for two GAPs. Another similarity with sparseness is that a value as extreme as *κ* = 0, that implies complete absence of non-genetic perturbations of the initial condition, is not able to stop the evolution of modularity after selection for GAPs *I* and *II*. One may think that these similarities would easily be explained if evolution under frequent perturbations produces an increase in sparseness and, consequently, on $Q_{P}^{N}$. This would require that the number of interactions were lower in populations evolving in the face of more perturbations. However, the expected number of interactions (30) is clearly in the bulk of the distribution of interactions in networks evolved under each of the three distinct values of *κ* that I tested. The mean ± SD number of interactions when *κ* equals 0, 0.025 and 0.05 is 28.65 ± 4.94, 30.58 ± 4.64 and 30.1 ± 4.34, respectively. These observations support that the effect that perturbation rate has on the evolution of modularity is not contingent on connectivity.

I also performed additional simulations in which selection favoured target GAPs *I* and *II* throughout evolution for 2 × 10^4^ generations. In these simulations, the value of *κ* changed from 0.01 to 0.05 after 10^4^ generations of evolution. Thus, networks were subject to more frequent perturbations of initial system states in the second stage of evolution. After evolution with the higher perturbation rate, the $Q_{P}^{N}$ score increased significantly (*W* = 97, 200; *p* < 2.2 × 10^−16^). Namely it increased from a mean value of 2.01 (SD = 0.92) to 2.64 (SD = 0.78). Together, the results that I present in this section support that a high perturbation rate, like sparseness, amplifies the effects on modularity that other mechanisms produce.

## Discussion

In this article I have addressed the role that sparseness plays in the evolution and consolidation of modules in gene regulatory networks that participate in development. The first possibility that I considered was that sparseness alone could explain modularity in such networks. This hypothesis requires gene regulatory networks to be as modular as random networks with the same number of interactions. My results suggest that this is not the case in developmental gene regulatory networks. A sample of 12 experimentally sustained regulatory networks involved in a wide variety of developmental processes suggests that such networks are more modular than expected for random networks with the same connectivity distribution. Whether this is a common property of other networks in biology remains an open question. A hint in this direction is that metabolic networks, represented as non-directed graphs, are also more modular than equally sparse random networks [76]. Here, however, my focus is on developmental regulatory networks. The results that I put forward suggest that factors other than sparseness must be invoked to explain modularity in developmental regulatory networks. One such factor may be selection for multiple gene activity phenotypes, as proposed previously [40].

Next I assessed whether sparseness collaborates with other factors to take modularity beyond the level expected in random networks. Because developmental regulatory networks have the ability to produce more than one GAP and also a modularity higher than that in random networks (Table 1), I decided to check how sparseness combines with selection to yield new GAPs. The analyses suggest that this selection regime increases modularity even when networks are very densely connected. Thus, sparseness is not absolutely required to make networks more modular than as predicted for random networks. Still, I found that sparseness amplifies the increase in modularity that selection for new activity phenotypes produces. Note that this effect does not depend on the random islands of high density that sparseness creates [50, 51]. After all, such analyses considered modularity with respect to the expectation in random networks with the same connectivity and referring to a specific partition *P* that selection favoured. Moreover, I found that my observations still hold after changes in the specifications of the model, in the identity of the target GAPs that selection favours and in parameter values (S1–S8 Figs).

That adaptation to produce new additional GAPs occurs less easily in dense networks suggest one reason why sparseness facilitates selection’s role in allocating interactions preferentially within modules. The mere abundance of inter-module connections may obstruct selection’s efforts to remove them. In addition, selection may also have lower incentives to remove interactions between modules in dense networks. The reason is that dense networks will also have more interactions within modules that counteract the pernicious effects of connections between modules.

The analyses that I present here support that, while sparseness facilitates the evolution of modularity, modularity can still evolve in non-sparse networks. One scenario that does suffice to obtain modules is that in which phenotypic optima vary in a modular manner, the so-called ‘modularly-varying goals’ scenario [38]. The ubiquity of environmental fluctuations raise the possibility that this scenario explains modularity in many organismal traits. For example, metabolic traits in bacteria that live in less stable environments tend to be more modular than those of bacteria in stable environments [39]. However, this may not be a general case. The metabolic networks in diverse fly or mammal species do not follow the same pattern [77]. Moreover, this scenario demands specifically ‘modular’ fluctuations, since fluctuations between random phenotypic optima do not promote modularity [38]. Another limitation is that the increase in modularity that this scenario produces is not stable. Once fluctuations stop, modularity drops abruptly [38].

Selection for new additional GAPs is a different option that also suffices to obtain modules in gene regulatory networks. In an evolutionary scale, the acquisition of new activity phenotypes has been a regular means to produce innovations across lineages [30, 41, 42]. Such new GAPs arise, for instance, when new cell types or body structures evolve. Indeed, sister cell types [41–43] and serially homologous structures [30, 44] share the activity of some genes, that allow us to recognize their relationship, but differ in the activity of other genes that underlie their distinct identities and functions. Therefore, nature frequently encounters the conditions that this scenario requires for the evolution of modularity in gene regulatory networks.

When selection combines genes that perform one function with other gene activity states, a new GAP emerges. This requires severing interactions that obstruct this new arrangement in gene activity. Thus, it is expected that modules reflect the selection process, as predicted previously [17]. Specifically, modules should group genes with activity states that are highly correlated in developmental end-states. This correlation will be positive or negative depending on whether selection rewards or punishes, respectively, their joint expression. For example, the algorithm by Leicht and Newman [55] identifies three modules in the Arabidopsis floral organ determination network [64]. The first module comprises meristem identity genes that determine whether a meristem is vegetative or gives rise to a flower. Such genes are active in one kind of meristem but not in the other. The second module concerns genes involved in deciding, within the flower, whether reproductive (carpels, stamens) or perianth (sepals, petals) organs are produced. Lastly, the third module concerns the decision between B-gene activity (petals, stamens) and no B-gene activity (sepals, carpels). Note that, in this framework, one module does not necessarily correspond to one function [78–80]. Two or more functions may be associated to the same module if genes assigned to distinct functions have correlated activity states in different parts of an organism or if the functions share a genetic basis.

That selection for multiple functions produces modular mechanisms may be valid even outside of the framework of gene regulatory networks and gene activity phenotypes. Modularity also appears in other kinds of systems where interactions between distinct sets of elements interfere and obstruct the performance of multiple beneficial functions. For example, modularity increases when selection favours networks that recognize two different patterns [45], RNA molecules that produce distinct structural units [4], gene networks that evolve to produce segments and differentiate them [81], and robots that grow and move [7] or that acquire steering and propulsion abilities [8].

Current data suggests that sparseness is widespread in gene regulatory networks. Its ubiquity does not seem to require an elaborate explanation. It stems naturally from the fact that it is easier for random genetic change to eliminate existing interactions than to create new ones [82]. The analyses that I put forward in this contribution suggest the existence of two distinct effects of sparseness in the evolution of modularity. Regarding the first effect, sparseness creates random islands where connection density is greater than in the rest of the network [50, 51]. The second is contingent on other factors, like some form of selection that makes interactions between specific sets of genes deleterious. I contend that the latter effect is more important in the evolution of gene regulatory networks for two main reasons. First, gene regulatory networks seem to be more modular than random networks with the same number of connections. Second, by amplifying the effects of selection, sparseness may facilitate adaptive evolution.

In this perspective, selection for new gene activity phenotypes may take outstanding importance. Besides producing stable increases in modularity [40], the ability to produce different GAPs seems associated with modularity in biological regulatory networks (Table 1). Moreover, the appearance of new gene activity phenotypes has been a frequent factor in the evolution of many organisms [31]. It may well have been a relevant factor in the evolution of modular gene regulatory networks.

## Supporting information

S1 Text Supplementary methods and analyses. (PDF)

S1 FigAnalyses considering all the populations, regardless successful adaptation.The data for this figure considers a network with the highest fitness in each population, irrespective of whether this fitness surpassed a threshold of 0.9. The results are qualitatively the same as those presented in the main text. (A) Modularity evolves after selection for an additional activity pattern. The evolutionary scenario is the same as that presented in Fig 3 in the main text (GAPs in Fig 3A). Mean ± SD $Q_{P}^{N}$ is lower in ancestral populations under selection for GAP *I* (−0.164 ± 1.009) than in populations evolved under selection for GAPs *I* and *II* (2.458 ± 0.988). Wilcoxon signed-rank test: *W* = 124, 950; *p* < 2 × 10^−16^. (B) Selection for two GAPs produces a greater increase in modularity in sparser networks. The evolutionary scenario is the same as that presented in Fig 4 in the main text (GAPs in Fig 3A). (C) The rate of perturbation of initial system states contributes to the increase in modularity due to selection for two GAPs. The evolutionary scenario is the same as that presented in Fig 5 in the main text (GAPs in Fig 3A).(PDF)

S2 FigAnalyses considering mean population values for populations where adaptation was successful.The data for this figure considers population averages in those populations where maximum fitness surpassed a threshold of 0.9. The results are qualitatively the same as those presented in the main text. (A) Modularity evolves after selection for an additional activity pattern. The evolutionary scenario is the same as that presented in Fig 3 in the main text (GAPs in Fig 3A). Mean ± SD $Q_{P}^{N}$ is lower in ancestral populations under selection for GAP *I* (−0.098 ± 0.895) than in populations evolved under selection for GAPs *I* and *II* (2.524 ± 0.773). Wilcoxon signed-rank test: *W* = 113, 030; *p* < 2 × 10^−16^. (B) Selection for two GAPs produces a greater increase in modularity in sparser networks. The evolutionary scenario is the same as that presented in Fig 4 in the main text (GAPs in Fig 3A). (C) The rate of perturbation of initial system states contributes to the increase in modularity due to selection for two GAPs. The evolutionary scenario is the same as that presented in Fig 5 in the main text (GAPs in Fig 3A).(PDF)

S3 FigEvolution of modularity and number of interactions with a different mutation setup.For the results in this figure, mutation was implemented in a different manner than in the rest of the paper. Here, a gene undergoing mutation acquired or lost an interaction with equal probabilities, regardless the number of regulators it has. The figure shows that, even when mutation is not biased to a particular number of regulators per gene, the number of interactions does not decrease beyond random expectation when modularity evolves after selection for an additional GAP. Results in panels A and B consider 125 populations that evolved 8,000 generations in the absence of selection. Panels C and D refer to 125 populations that evolved in a similar scenario as populations described in Fig 3 in the main text. That is, they evolved first under selection for a single GAP (GAP *I* in Fig 3A) and then under selection for two GAPs (*I* and *II* in Fig 3A). (A) Distribution of the number of regulators per gene after 8,000 generations of neutral evolution. The figure is coherent with a uniform distribution for the number of regulators per gene with this mutation set up. (B) Distribution of the number of interactions per network. The mean number of interactions per network is 49.592 (SD = 10.21). This expected value is thus close to the theoretical expectation of 50 interactions per network. (C) $Q_{P}^{N}$ increases significantly after selection for an additional activity pattern. Specifically, it increases from −0.077 ± 0.98 to 1.875 ± 0.865 (*W* = 7, 778; *p* < 2.2 × 10^−16^). (D) The number of interactions after selection for either one or two GAPs is not lower than in the absence of selection (compare to panel B). The mean ± SD number of interactions is 56.42 ± 8.88 after selection for GAP *I* and 59.67 ± 8.5 after selection for GAPs *I* and *II*.(PDF)

S4 FigSparseness contributes to modularity after selection for an additional GAP in larger networks.For the simulations in this figure the parameters that I used were the following: *N* = 16, *μ* = 0.02, *κ* = 0.03, *S* = 0.8, *K* = 50/*T*. (A) Target GAPs *X* and *Y*. Genes 1-8 are grouped in set $A^{\prime }$ and genes 9-16 are grouped in set $B^{\prime }$. Note that genes in set $A^{\prime }$ have the same activity state in both target GAPs and genes in set $B^{\prime }$ have a different activity state in both target GAPs. (B) Sparseness contributes to modularity after selection for an additional GAP. The figure compares results for 50 populations evolved under a propensity to gain interactions *γ* = 3/16 (48 expected interactions) and 50 populations evolved under a propensity to gain interactions *γ* = 5/16 (80 expected interactions). Network populations evolved first under selection to yield target GAP *X* for 1,500 generations. In a second stage that lasted 7,500 generations, selection favoured networks that produce GAPs *X* and *Y* from different initial system states.(PDF)

S5 FigSparseness contributes to modularity after selection for an additional GAP under a different model of network dynamics.For the simulations in this figure the value of *θ*_*i*_ is set to 0 for all genes. Therefore, network dynamics is given by $s_{i}^{t+1}=\sigma_{i}[\sum_{j=1}^{N}g_{ij}s_{j}^{t}]$, where the function *σ*_*i*_(*x*) equals 1 when *x* > 0, it equals $s_{i}^{t}$ when *x* = 0 and it equals 0 when *x* < 0. The figure compares results for 125 populations evolved under a propensity to gain interactions *γ* = 0.2 (20 expected interactions) and 125 populations evolved under a propensity to gain interactions *γ* = 0.4 (40 expected interactions). The evolutionary scenario is the same as that presented in Figs 3 and 4 in the main text (GAPs in Fig 3A), in which populations evolved first under selection for a single GAP (GAP *I*) for 2,000 generations and then under selection for two GAPs (GAPs *I* and *II*) for 8,000 generations. The figure shows that, also with this model, selection for two GAPs produces a greater increase in modularity in sparser networks.(PDF)

S6 FigSparseness contributes to modularity after selection for an additional GAP when the entries of genotype matrices are described by continuous variables.For the simulations in this figure, the probability that a regulation of gene *i* is lost equals $\mu \left(1-\gamma \right)\frac{R_{i}}{N}$. The probability that an interaction is acquired is $\mu \gamma \frac{N-R_{i}}{N}$. In this case, the new weight of the interaction is taken from an N(0,1) distribution. The probability that the weight of an interaction is modified by mutation is $\mu \gamma \frac{R_{i}}{N}$. When an existing interaction is modified, the new weight is taken from an N(0,1) distribution, but its sign is forced to be the same that it had before mutation. The figure compares results for 125 populations evolved under a propensity to gain interactions *γ* = 0.2 (20 expected interactions) and 125 populations evolved under a propensity to gain interactions *γ* = 0.4 (40 expected interactions). The evolutionary scenario is the same as that presented in Figs 3 and 4 in the main text (GAPs in Fig 3A), in which populations evolved first under selection for a single GAP (GAP *I*) for 2,000 generations and then under selection for two GAPs (GAPs *I* and *II*) for 8,000 generations. The figure shows that, also with this model, selection for two GAPs produces a greater increase in modularity in sparser networks.(PDF)

S7 FigSparseness contributes to modularity after selection for two GAPs that differ in the activity of three genes.(A) Target GAPs *I* and *IV*. Genes 1-7 are grouped in set C and genes 8-10 are grouped in set D. Note that genes in set C have the same activity state in both target GAPs. In contrast, the three genes in set D have a different activity state in both target GAPs. Networks evolve in a first stage under selection to produce target GAP *I*. In a second stage, selection favours networks that produce target GAPs *I* and *IV* from distinct initial system states. (B) After selection for both target GAPs *I* and *IV*, interactions occur mainly either between genes in set C or between genes in set D. (C) Selection for two GAPs produces a greater increase in modularity in sparser networks.(PDF)

S8 FigSparseness contributes to modularity after selection for two GAPs that differ in the activity of seven genes.(A) Target GAPs *I* and *V*. Genes 1-3 are grouped in set E and genes 4-10 are grouped in set F. Note that genes in set E have the same activity state in both target GAPs. In contrast, the seven genes in set F have a different activity state in both target GAPs. Networks evolve in a first stage under selection to produce target GAP *I*. In a second stage, selection favours networks that produce target GAPs *I* and *V* from distinct initial system states. (B) After selection for both target GAPs *I* and *V*, interactions occur mainly either between genes in set E or between genes in set F. (C) Selection for two GAPs produces a greater increase in modularity in sparser networks.(PDF)
