# Supplementary material for: On the role of sparseness in the evolution of modularity in gene regulatory networks
Source: PLoS Comput Biol. 2018 May 18;14(5):e1006172. doi: 10.1371/journal.pcbi.1006172 (PMC5979046; doi:10.1371/journal.pcbi.1006172)
Supplement: S1 Text — (PDF) [file pcbi.1006172.s001.pdf]

S1 Text. Supplementary methods and analyses for '*On the role of sparseness in the evolution of modularity in gene regulatory networks*'

Carlos Espinosa-Soto

Contents

S1T.1 Supplementary methods **1**
S1T.1.1 Properties of the model for network dynamics 1
S1T.1.1.1 Distinct equivalent formulations of the model for network dynamics 1
S1T.1.1.2 Generalization to other single GAPs 2
S1T.1.1.3 Generalization to other pairs of GAPs 3
S1T.1.2 Distance between GAPs 4
S1T.1.3 Mutation 6

S1T.2 Supplementary analyses **6**
S1T.2.1 Duration of evolutionary simulations 6
S1T.2.2 Evolution of the raw modularity score Q^{opt} 7

S1T.1 Supplementary methods

S1T.1.1 Properties of the model for network dynamics

S1T.1.1.1 Distinct equivalent formulations of the model for network dynamics

The model formulation that I present in the main text considers that the activity state of a gene i may be $s_i^t = 1$ if i is active and $s_i^t = 0$ if i is inactive. Dynamics is guided by gene interactions summarized in a matrix $\mathbf{G}$. In this matrix, a positive entry $g_{ij} > 0$ means that gene j promotes the activation of gene i and a negative entry $g_{ij} < 0$ that gene j inhibits the expression of gene i. An entry $g_{ij} = 0$ means that the activity of gene j has no direct effect on gene i. The main text explains that genes change their activity state according to:

$$s_i^{t+1} = \sigma_i \left[\sum_{j=1}^{N} g_{ij} s_j^t - \theta_i \right] \quad \text{, where :} \quad \sigma_i(x) = \begin{cases} 1, & \text{if } x > 0 \\ s_i^t, & \text{if } x = 0 \\ 0, & \text{if } x < 0 \end{cases} \quad \text{, and :} \quad \theta_i = \frac{\sum_{j=1}^{N} g_{ij}}{2} \tag{1}$$

The model just mentioned can be rephrased in a distinct formulation. In this alternative version, an active gene i has an activity state equal to +1 and the activity state of an inactive gene is -1. Dynamics is given by:

$$s_i^{t+1} = \sigma_i \left[\sum_{j=1}^{N} g_{ij} s_j^t \right] \quad \text{, where :} \quad \sigma_i(x) = \begin{cases} 1, & \text{if } x > 0 \\ s_i^t, & \text{if } x = 0 \\ -1, & \text{if } x < 0 \end{cases} \tag{2}$$

The latter formulation is a version of a model first put forward by A. Wagner [1, 2]. Because the two formulations are equivalent, a gene responds in the same way in any of them, either becoming active or inactive, when exposed to the same combination of active/inactive regulators.

S1T.1.1.2 Generalization to other single GAPs

The model has important attributes that make it specially useful for the study of gene regulatory networks. One of this attributes concerns properties of ensembles of networks that build one arbitrary stationary GAP A from a specified initial system state $\mathbf{a}^0$ [3]. Ciliberti et al. found that an important parameter is the number d of genes with different activity states in GAP A and initial system state $\mathbf{a}^0$. Consider a second arbitrary stationary GAP B and another initial system state $\mathbf{b}^0$, that also differ in the activity of d genes. For networks that build GAP A from the initial condition $\mathbf{a}^0$, there is a one-to-one transformation into networks that build GAP B from $\mathbf{b}^0$.

In brief, the transformation of a network, with a matrix representation $\mathbf{G}_A$, that yields A from $\mathbf{a}^0$ to an equivalent network $\mathbf{G}_B$ that produces B from $\mathbf{b}^0$ involves the following steps [3]:

- Rearrange the order in which genes appear in $\mathbf{a}^0$ and A so that genes with different activity state in the initial state and the stationary GAP appear in the same position as genes with different activity state in the pair $\mathbf{b}^0$ and B. We may call $\mathbf{a}^{0'}$ and A' such rearranged initial state and GAP, respectively. Rearrange the columns and rows of $\mathbf{G}_A$ accordingly to obtain a new matrix $\mathbf{G}'_A$. The rearrangement determines the correspondence between genes in the original network and genes in the new network.

- For every gene that appears in position i in the rearranged GAP A' and that has a different activity state as the i-th entry in GAP B, multiply by -1 all the entries in the i-th column and in the i-th row of the rearranged matrix $\mathbf{G}'_A$.

The original and the new networks have the same dynamic and variational properties. Consider the transformation that we just performed from a network $\mathbf{G}_A$ that produces A from $\mathbf{a}^0$ to a network $\mathbf{G}_B$ that produces B from $\mathbf{b}^0$. Consider as well, for example, an arbitrary initial system state $\mathbf{x}^0$ that leads to A in the original network and that differs from A in the activity of k genes. Then, an initial system state $\mathbf{y}^0$ exists in the new network that leads to B and that differs from B in the activity of the k genes that correspond (according to the rearrangement that we performed) to those that differ between $\mathbf{x}^0$ and A. Moreover, for every mutation that in $\mathbf{G}_A$ produces an effect relative to A there is a corresponding mutation that produces the same effect in $\mathbf{G}_B$ relative to B.

We can perform the same transformation to any network in the ensemble of networks that produce A from $\mathbf{a}^0$, as explained by Ciliberti et al. [3]. Remember that B and $\mathbf{b}^0$, that we used to perform the transformation, were arbitrary, with the only restriction of having the same number of differences d as $\mathbf{a}^0$ and A. Therefore, observations that are valid for the set of networks that produce A from $\mathbf{a}^0$ are also valid for the set of networks that produce any stationary GAP from an initial state that differs in the activity of d genes.

Now, let's examine the properties of the model in the context of the evolutionary simulations presented in this study. Consider simulations where networks evolve, as described in Methods in the main text, under selection to maintain a single stationary GAP A. The default initial condition, in the absence of perturbations is A itself ($d = 0$). Following the procedure described above, we can find a one-to-one transformation from networks that maintain A to networks that maintain an arbitrary stationary GAP B. Perturbations of the initial condition do not invalidate the relationship between a network $\mathbf{G}_A$ that maintains A and a network $\mathbf{G}_B$ that results from the transformation of $\mathbf{G}_A$ and that produces B. The probability of obtaining an initial

condition that differs from A in the activity of k genes for $\mathbf{G}_A$ follows the same distribution as the probability of obtaining for $\mathbf{G}_B$ an initial condition that differs from B in the activity of the corresponding k genes. Moreover, it is specially relevant for the simulation of evolutionary processes that the effect of a mutation in network $\mathbf{G}_A$ relative to GAP A is preserved in the network that results from the one-to-one transformation $\mathbf{G}_B$, but relative to GAP B. Thus, in this set up, what is true for those scenarios in which A evolves is also true for scenarios where any other stationary GAP takes the place of A.

S1T.1.1.3 Generalization to other pairs of GAPs

Many of the evolutionary simulations that I present involve selection for two stationary GAPs that we may call A and B. I will call h to the number of genes with different activity states in GAPs A and B. Consider a network that is able to maintain the two stationary GAPs (*i.e.* $d = 0$ for both GAPs). Consider as well two other stationary GAPs, C and D, that also differ in the activity of h genes. There is also a one-to-one transformation from networks that maintain A and B to networks that maintain C and D. Starting from a network, with a matrix representation $\mathbf{G}_{AB}$, that produces GAPs A and B, the transformation involves the following steps:

- Rearrange the order in which genes appear in A and B so that the h genes with different activity state appear in the same position as those with different activity state in the pair of GAPs C and D. Rearrange the columns and rows of $\mathbf{G}_{AB}$ accordingly. I will refer to the rearranged GAPs and matrix as A', B' and $\mathbf{G}'_{AB}$. That after this step $\mathbf{G}'_{AB}$ has the same properties relative to A' and B' as $\mathbf{G}_{AB}$ relative to A and B is obvious since we are merely listing the genes in a different order in the matrix and in the GAPs. This rearrangement defines which gene in the original network corresponds to which gene in the new network.

- For every gene that appears in position i in the rearranged GAP A' and that has a different activity state as the i-th entry in GAP C, multiply by -1 all the entries in the i-th column and in the i-th row of the rearranged matrix $\mathbf{G}'_{AB}$.

Multiplying row i's entries by -1 guarantees that all the factors that kept gene i in an activity state opposed to the one it should have in GAP C now keep it in the right activity state with the same strength. Multiplying column i's entries by -1 guarantees that the effects that gene i had on other network genes before changing the i-th row will have the same sign and magnitude after changing that row. Thus the sequence of changes that other genes follow is not perturbed. Consequently, the new matrix, $\mathbf{G}_{CD}$, maintains GAP C, just like the original network maintained GAP A.

Now let's see some of the properties of the transformation that we have just performed. Consider an arbitrary initial condition in the original system $\mathbf{x}^0$. This initial condition will differ from GAP A in the activity of some genes, that we may group in a set V. For the new network, we may define another set V' that groups together the genes that correspond to genes in V, according to the rearrangement that we performed in the first step of the transformation. We thus can build an initial system state $\mathbf{y}^0$ for the new network by taking GAP C and changing the activity state of those genes in V'. If we subject $\mathbf{x}^0$ to dynamics driven by the original network $\mathbf{G}_{AB}$ and $\mathbf{y}^0$ to dynamics driven by $\mathbf{G}_{CD}$ we will observe that for every gene that changes its activity state in the original network, the corresponding gene also changes its activity state in the new network. This pattern follows throughout the two dynamic trajectories. Indeed, if $\mathbf{G}_{AB}$ leads to GAP A when network dynamics start from $\mathbf{x}^0$, then $\mathbf{G}_{CD}$ will lead to GAP C when dynamics start from $\mathbf{y}^0$. Moreover, if in the original system $\mathbf{x}^0$ leads to GAP B, then $\mathbf{y}^0$ will lead to GAP D. Therefore, the new network, besides producing C, also yields D just like the original network produced A and B.

The transformation that we performed preserves all the properties that the original network has with respect to GAPs A and B, but relative to GAPs C and D. Importantly, this relationship between $\mathbf{G}_{AB}$ and $\mathbf{G}_{CD}$ does not only pertain to variations in initial conditions, but also to changes in gene interactions. If a

mutation in $\mathbf{G}_{AB}$ causes it to produce, instead of A, a GAP that differs from A in the activity of k genes, then the corresponding mutation in $\mathbf{G}_{CD}$ will cause it to produce a GAP that differs from C in the activity of the k corresponding genes. Taken together, this means that the new network preserves the same dynamic and variational properties of the original network $\mathbf{G}_{AB}$. Therefore, with the set up that I use, observations that are valid for scenarios under selection for a specific pair of GAPs that differ in the activity of h genes are also valid in scenarios where selection favours in the same manner any pair of GAPs with the same number of differences between them.

S1T.1.2 Distance between GAPs

In order to evaluate a network's fitness, it is necessary to compare how similar is the GAP that each developmental trajectory produces to a target GAP that is assumed to produce a biological function optimally (see subsection 'Evaluation of a network's fitness' in Methods).

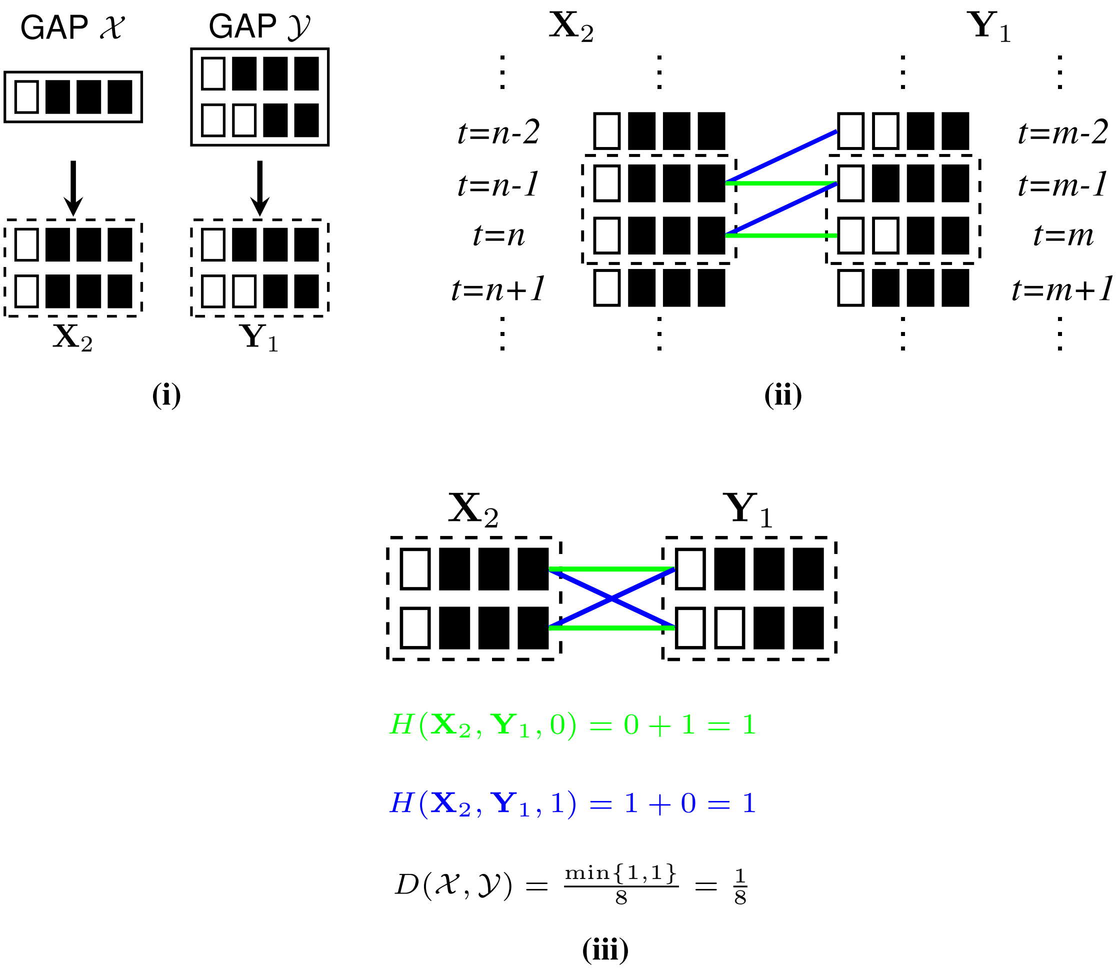

Fig A. Measuring the distance between GAPs with unequal periods. (i) Building versions of GAPs with the same number of rows. (ii) Different pairing between system states that preserve the order in which they appear. (iii) Assessing the number of differences for each of the possible associations and evaluating the distance between GAPs.

In the more general case, two GAPs $\mathcal{X}$ and $\mathcal{Y}$ may not have the same period $k_{\mathcal{X}} \neq k_{\mathcal{Y}}$. That is, there is a different number of system states that are repeated indefinitely in each GAP. Fig A(i) shows an example in which GAP $\mathcal{X}$ is stationary ($k_{\mathcal{X}} = 1$) and GAP $\mathcal{Y}$ is a limit cycle ($k_{\mathcal{Y}} = 2$).

In order to compare two GAPs of unequal sizes, one may describe a GAP $\mathcal{X}$ repeating any number $u \in \mathbb{Z}^+$ of times its $k_\mathcal{X}$ system states. I refer to the resulting row augmented matrix of size $(k_\mathcal{X} u) \times N$ as $\mathbf{X}_u$ (Fig A(i)). One may do the same for the other GAP $\mathcal{Y}$, thus building a row augmented matrix $\mathbf{Y}_v$ of size $(k_\mathcal{Y} v) \times N$. Hence, to measure the distance between the two GAPs I first find u and v values such that the sizes of the row augmented matrices $\mathbf{X}_u$ and $\mathbf{Y}_v$ are the same ($k_\mathcal{X} u = k_\mathcal{Y} v$). This step allows comparing versions of the network GAP $\mathcal{Y}$ and GAP $\mathcal{X}$ with the same number of entries, even if their period does not coincide. In the example of Fig A(i), $u = 2$ and $v = 1$.

Once one has versions of the GAPs $\mathbf{X}_u$ and $\mathbf{Y}_v$ with the same number of rows $R = k_\mathcal{X} u = k_\mathcal{Y} v$, the next step is to evaluate how different they are. To do so, it is required to find which row of $\mathbf{X}_u$ corresponds to which row of $\mathbf{Y}_v$. There are R forms of pairing the system states in the two GAPs without disturbing the order in which they appear (coloured lines in Figs A(ii),(iii)). Each of them associates the i-th system state in one GAP to the system state that in the second GAP appears in row $i + j \pmod R$, with $j \in \{0, 1, \dots, R - 1\}$. For each manner j of pairing system states, $H(\mathbf{X}_u, \mathbf{Y}_v, j)$ is the sum of the number of differences between corresponding rows (coloured equations in Fig A(iii)). Among all R distinct forms of relating system states, I pick the one in which $H(\mathbf{X}_u, \mathbf{Y}_v, j)$ has the lowest value (Fig A(iii)). Doing this allows to recognise similar GAPs that are described starting from different steps of the cycle. Finally, the result is divided by the total number of compared entries to get a distance $0 \le D(\mathcal{X}, \mathcal{Y}) \le 1$:

$$D(\mathcal{X}, \mathcal{Y}) = \frac{\min(\{H(\mathbf{X}_u, \mathbf{Y}_v, j) : j = 0, 1, \dots, k_\mathcal{X} u - 1\})}{k_\mathcal{X} u N} \quad (3)$$

When the GAPs that will be compared have the same period, then it is not necessary to build any row augmented matrix (see example in Fig B). When $k_\mathcal{X} = k_\mathcal{Y}$, then $u = v = 1$ and the distance between the two GAPs is merely:

$$D(\mathcal{X}, \mathcal{Y}) = \frac{\min(\{H(\mathcal{X}, \mathcal{Y}, j) : j = 0, 1, \dots, k_\mathcal{X} - 1\})}{k_\mathcal{X} N} \quad (4)$$

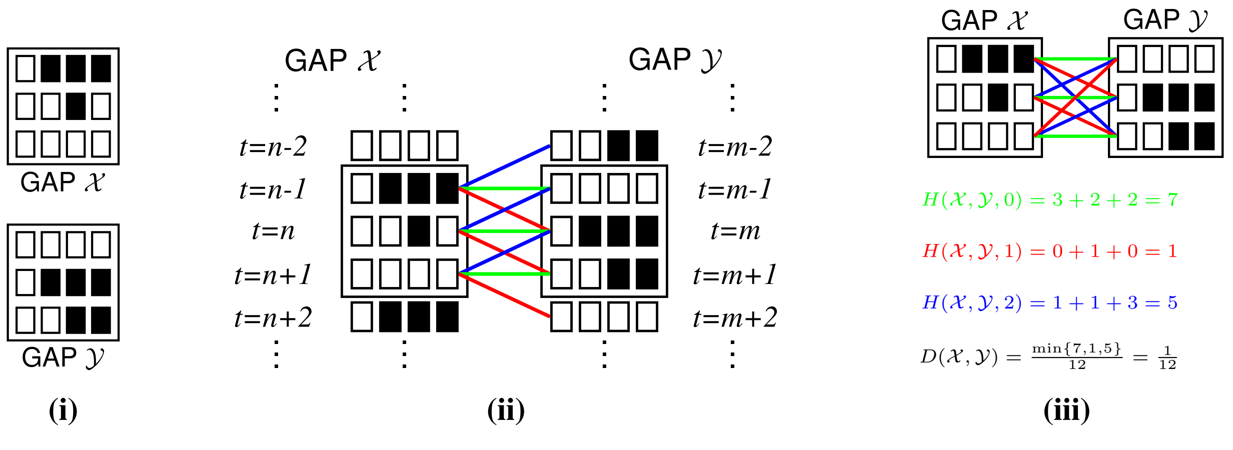

Fig B. Measuring the distance between GAPs with equal periods. (i) GAPs with the same number of rows. (ii) Different pairing between system states that preserve the order in which they appear. (iii) Assessing the number of differences for each of the possible associations and evaluating the distance between GAPs.

This general case simplifies to an even more intuitive situation when both GAPs are stationary ($k_\mathcal{X} = k_\mathcal{Y} = 1$). In this case there is only one possible association between the single rows in each GAP. Thus, the distance between the two GAPs is defined by the fraction of genes with different activity states:

$$D(\mathcal{X},\mathcal{Y}) = \frac{H(\mathcal{X},\mathcal{Y})}{N} \quad (5)$$

S1T.1.3 Mutation

Mutation merely affects the number of regulators that a gene has. Thus, a mutation changes an entry in the matrix $\mathbf{G}$ that describes gene interactions. Other kinds of mutation, like gene duplication, are not considered in this contribution. A gene i acquires a new regulatory interaction with probability $\mu\gamma\frac{N-R_i}{N}$, where μ is the mutation rate per gene, γ is the propensity to gain interactions and R_i is the number of genes that regulate gene i. In this case, the new regulator is chosen with uniform probability among the $N - R_i$ genes that did not regulate gene i before mutation and the new interaction is equally likely to be positive or negative. The probability that a gene loses a regulatory interaction equals $\mu(1-\gamma)\frac{R_i}{N}$. The interaction that is lost is then picked at random among those that regulate gene i. Note that the probability of losing an interaction increases with R_i, while the probability of acquiring an interaction decreases. Also note that these two probabilities are equal when $R_i = N\gamma$. Therefore, mutation tends to pull the number of regulators to $N\gamma$, the expected number of regulators per gene. Thus, one may define how sparse or dense networks tend to be by tuning γ.

The setup that I use to implement mutation assumes that the regulatory region of a gene with many distinct transcriptional regulators contains more transcription factor binding sequences than a gene with few regulators. Thus, the probability that a random mutation wrecks a regulatory interaction increases with the number of regulators. The setup also assumes that new functional transcription factor binding sequences appear without disrupting those that already exist. Hence, a regulatory region with few functional binding sequences will offer more opportunities for the appearance of new such sequences. The value of γ, that modulates how easy it is to gain or lose interactions, may be associated to biophysical parameters as specificity or the length of transcription factor binding sequences. These parameters affect the number of distinct DNA sequences that yield meaningful interactions with transcription factors [4, 5].

S1T.2 Supplementary analyses

S1T.2.1 Duration of evolutionary simulations

The possibility exists that, for some of the evolutionary scenarios addressed in the main text, modularity or other network properties may still be evolving when the simulation stops. If this were the case, the potential of an evolutionary scenario to produce an increase in modularity may not be correctly assessed. One way that I used to avoid this potential problem was to consider a duration of the evolutionary process that surpasses by far the number of generations that are typically required to achieve maximal fitness. For example, the figure below (Fig C) shows how maximal fitness changes in populations that evolve in simulations that consist of two distinct stages. In the first stage, that lasts 2,000 generations, selection favours the construction of a single GAP (GAP I in Fig 3A in the main text). In the remaining 8,000 generations, selection favours networks that can produce two different GAPs (GAPs I and II in Fig 3A in the main text) from different initial conditions. The figure shows that more than 75% of the populations have reached a fitness equal to one after 3,000 generations in the second stage (at generation 5,000), many generations before the simulation finishes. Another strategy was limiting my analyses to those populations that have adapted successfully, as they achieve a fitness that exceeds a threshold of 0.9. Nevertheless, one may still doubt whether taking these measures was sufficient. I contend that this was the case for reasons explained in the next paragraph.

In case it existed, the problem of modularity still evolving by the end of the simulation would be more severe in evolutionary scenarios where adaptation is more difficult and slow. The scenario where adaptation was clearly less frequent than in all other scenarios was when there was selection for two GAPs and

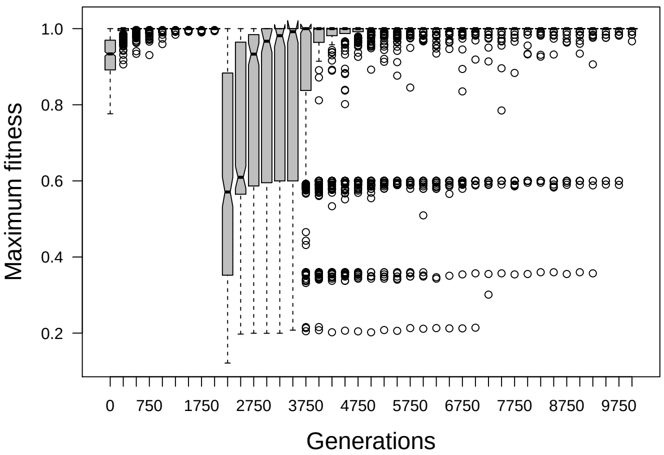

Fig C. Evolution of maximum fitness in populations that successfully adapted. From generation 0 to 2000 there is selection for a single GAP. Afterwards, there is selection for two GAPs. Default parameter values. Each box and the dots directly above and below it presents the distribution of maximum fitness at generation t. The waist in each box indicates the median value at that time. The upper and lower border in each box indicate the third and first quartile, respectively. Thus, the lower border in a gray box indicates the fitness value that is surpassed by 75% of the data points. Dots denote outlier fitness values.

the propensity to gain interactions was unrealistically high ($\gamma = 0.7$, described in section 'The effects of sparseness on the evolution of modularity' in the main text). Thus, I ran other simulations where I allowed evolution for 28,000 generations in the 'difficult' scenario instead of only 8,000 generations. I found that, as expected, many more populations adapt successfully in the long evolutionary simulations. Notwithstanding, the differences were very small and not significant when I compared the modularity (Q_P^N) in successfully adapted populations in short and long simulations. Specifically, mean $\pm$ SD Q_P^N was 1.292 ± 0.964 and 1.336 ± 0.902 for short and long simulations, respectively. The differences were not significant, according to a Mann-Whitney U test ($U = 66,490$; $p = 0.503$). In sum, these results suggest that, even in such a difficult scenario, evolution of modularity had come to a halt in successfully adapted populations.

S1T.2.2 Evolution of the raw modularity score Q^{opt}

Several previous studies have approached the issue of evolution of modularity using the modularity score that results from the application of algorithms that find the partition that maximizes intra-group connection density [e.g. 6–10]. I refer to this score as Q^{opt}. As explained in the main text, this score does not separate the effects that sparseness alone has on modularity, which was the starting point of most of the analyses that I present. Notwithstanding, here I refer to results that show how Q^{opt} changes in the evolutionary scenarios that I considered in the main text. The reader may find useful these analyses for comparisons to studies that use this score.

First I consider the same scenario as in Fig 3 in the main text. In this scenario, populations first evolve under selection for a single target GAP (GAP I in Fig 3A in the main text). In a second stage, selection favours networks that produce two distinct GAPs from different initial system states (GAPs I and II in Fig 3A). Previous research had already shown that Q^{opt} increases significantly when networks evolve new additional gene activity patterns [11]. With the parameters that I used in this paper, networks that evolved under selection for a single GAP had a mean $\pm$ SD Q^{opt} equal to 0.192 ± 0.043. After selection for two GAPs Q^{opt} was 0.235 ± 0.059. This increase was statistically significant ($W = 90,392$;$p < 2.2 \times 10^{-16}$; Fig D).

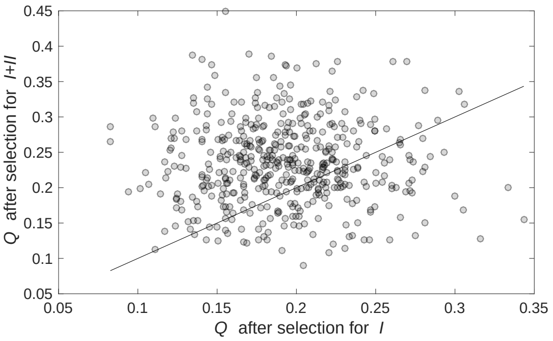

Fig D. Evolution of the raw modularity score Q^{opt}. Evolution of Q^{opt} due to selection for new additional GAPs. The scenario and parameters used for simulations in this panel is the same as in Fig 3 in the main text.

When assessing distinct values for the propensity to gain interactions γ the results for Q^{opt} are different as those reported in the main text. This difference is expected because Q^{opt} does not discard the effects that sparseness alone has on modularity. Thus, as networks become more connected, the value of Q^{opt} decreases both in ancestral networks that produce a single GAP and in evolved networks with the capacity to produce two GAPs from different initial system states (Fig E(ii)).

Another expected discrepancy occurs when networks are under selection for a single GAP, but they evolve first with a high propensity to gain interactions $\gamma = 0.4$ and in a second stage the value of γ is halved. Because Q^{opt} does not discard the effects that sparseness has on modularity, Q^{opt} increases significantly after networks become sparser ($W = 121, 110$; $p < 2.2 \times 10^{-16}$; Fig E(i)). Specifically, it increases from 0.152 ± 0.038 to 0.232 ± 0.053. In contrast, when the effects of sparseness alone are removed, as in the main text, the modularity score did not increase significantly.

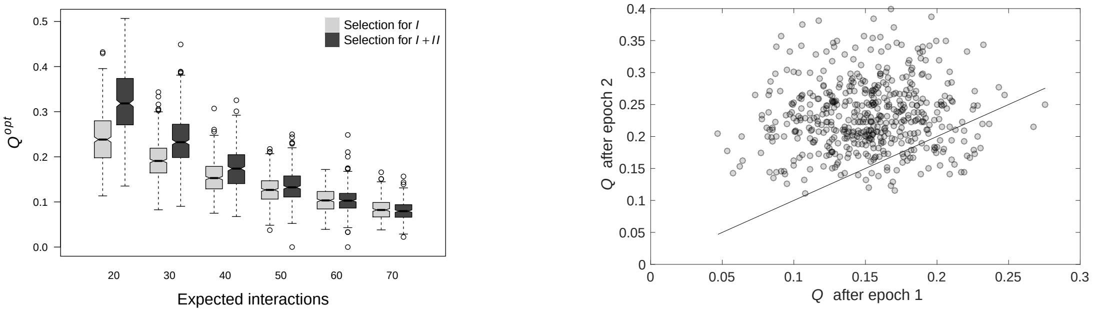

(i) **(ii)**

Fig E. Evolution of the raw modularity score Q^{opt} in sparse and non-sparse networks. (i) Q^{opt} increases in sparse networks both in networks evolved under selection for a single GAP and in networks selected to produce two GAPs. The scenario and parameters used for simulations in this panel is the same as in Fig 4 in the main text. (ii) Q^{opt} increases after networks become sparser. Throughout evolution networks are under selection for a single GAP. In a first stage, the propensity to gain interactions, γ, equals 0.4. In a second stage, γ equals 0.2.

References

1. Wagner A. Evolution of gene networks by gene duplications: A mathematical model and its implications on genome organization. Proc Natl Acad Sci USA. 1994;91(10):4387–91. doi:10.1073/pnas.91.10.4387.

2. Wagner A. Does Evolutionary Plasticity Evolve? Evolution (N Y). 1996;50:1008–1023. doi:10.2307/2410642.

3. Ciliberti S, Martin OC, Wagner A. Robustness can evolve gradually in complex regulatory gene networks with varying topology. PLoS Comput Biol. 2007;3(2):e15. doi:10.1371/journal.pcbi.0030015.

4. Sengupta AM, Djordjevic M, Shraiman BI. Specificity and robustness in transcription control networks. Proc Natl Acad Sci USA. 2002;99(4):2072–2077. doi:10.1073/pnas.022388499.

5. Tuğrul M, Paixão T, Barton NH, Tkačik G. Dynamics of transcription factor binding site evolution. PLoS Genet. 2015;11(11):e1005639. doi:10.1371/journal.pgen.1005639.

6. Livingston N, Bernatskiy A, Livingston K, Smith ML, Schwarz J, Bongard JC, et al. Modularity and sparsity: Evolution of neural net controllers in physically embodied robots. Front Robot AI. 2016;3:75. doi:10.3389/frobt.2016.00075.

7. Ellefsen KO, Mouret JB, Clune J. Neural Modularity Helps Organisms Evolve to Learn New Skills without Forgetting Old Skills. PLoS Comput Biol. 2015;11(4):e1004128. doi:10.1371/journal.pcbi.1004128.

8. Takemoto K. Habitat variability does not generally promote metabolic network modularity in flies and mammals. Biosystems. 2016;139:46–54. doi:10.1016/j.biosystems.2015.12.004.

9. Mengistu H, Huizinga J, Mouret JB, Clune J. The evolutionary origins of hierarchy. PLoS Comput Biol. 2016;12(6):e1004829. doi:10.1371/journal.pcbi.1004829.

10. Clune J, Mouret JB, Lipson H. The evolutionary origins of modularity. Proc R Soc Lond B Biol Sci. 2013;280(1755):20122863. doi:10.1098/rspb.2012.2863.

11. Espinosa-Soto C, Wagner A. Specialization can drive the evolution of modularity. PLoS Comput Biol. 2010;6(3):e1000719. doi:10.1371/journal.pcbi.1000719.
