# Supplementary figures and images for: On the role of sparseness in the evolution of modularity in gene regulatory networks
Source: PLoS Comput Biol. 2018 May 18;14(5):e1006172. doi: 10.1371/journal.pcbi.1006172 (PMC5979046; doi:10.1371/journal.pcbi.1006172)

**A**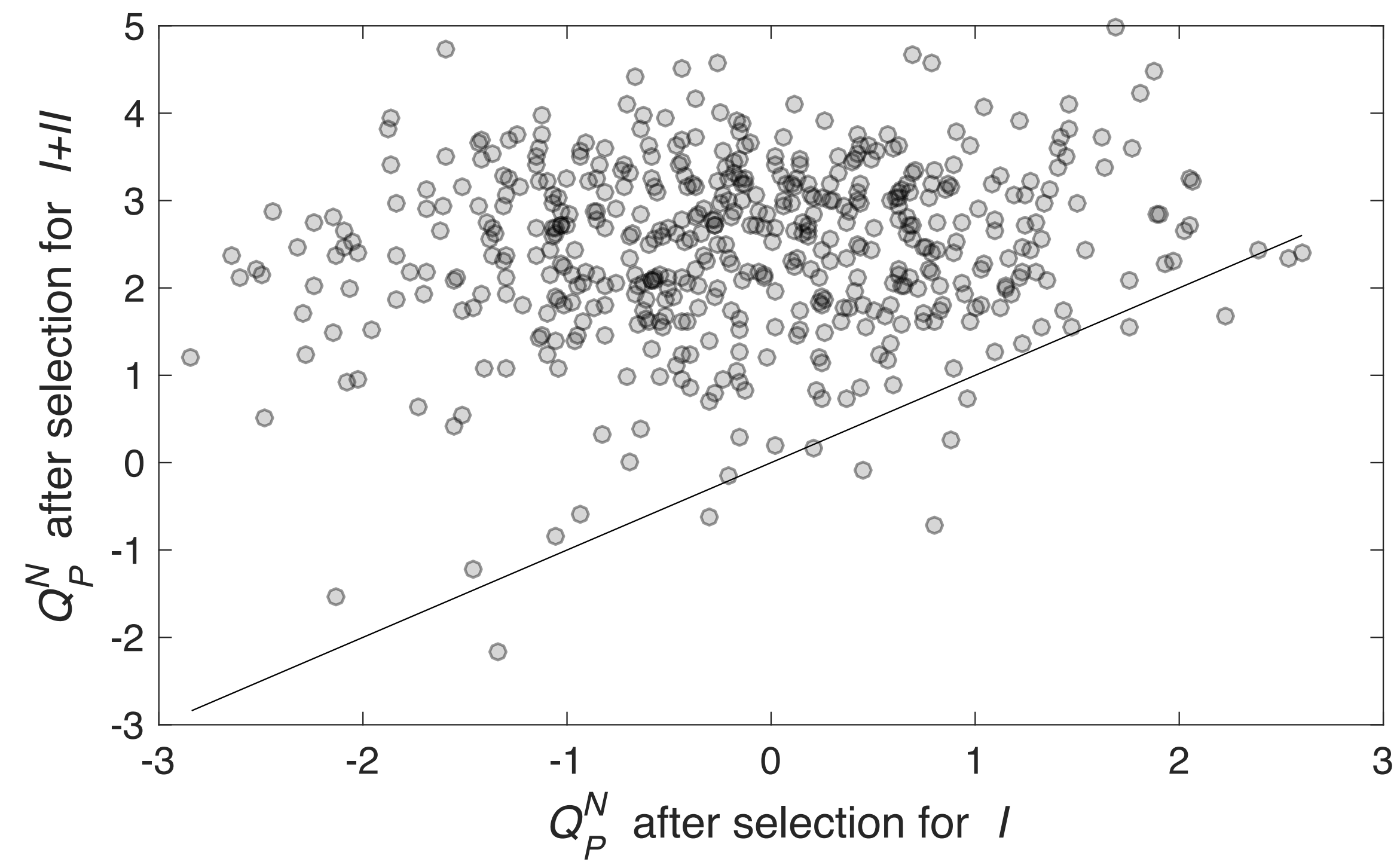**B**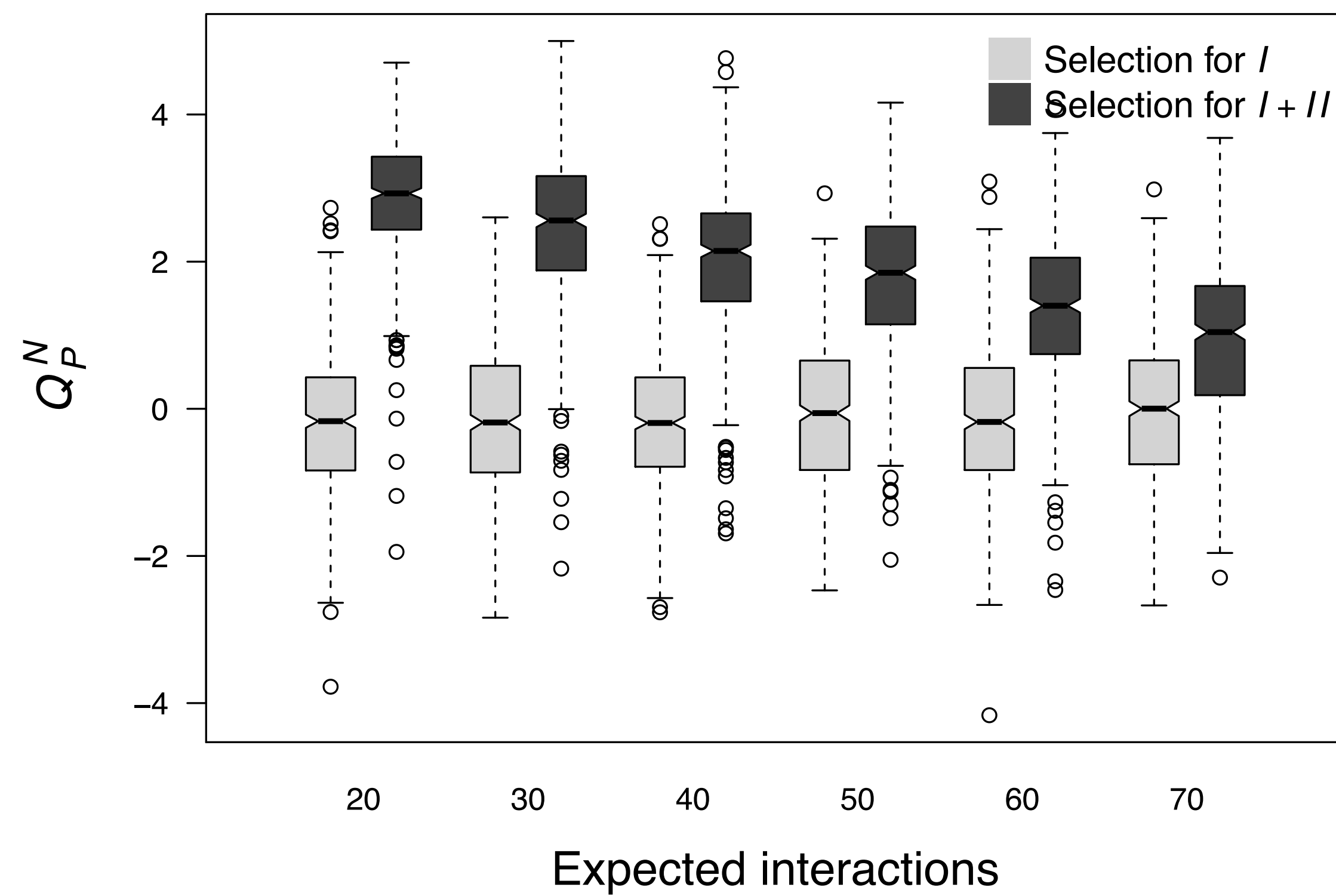**C**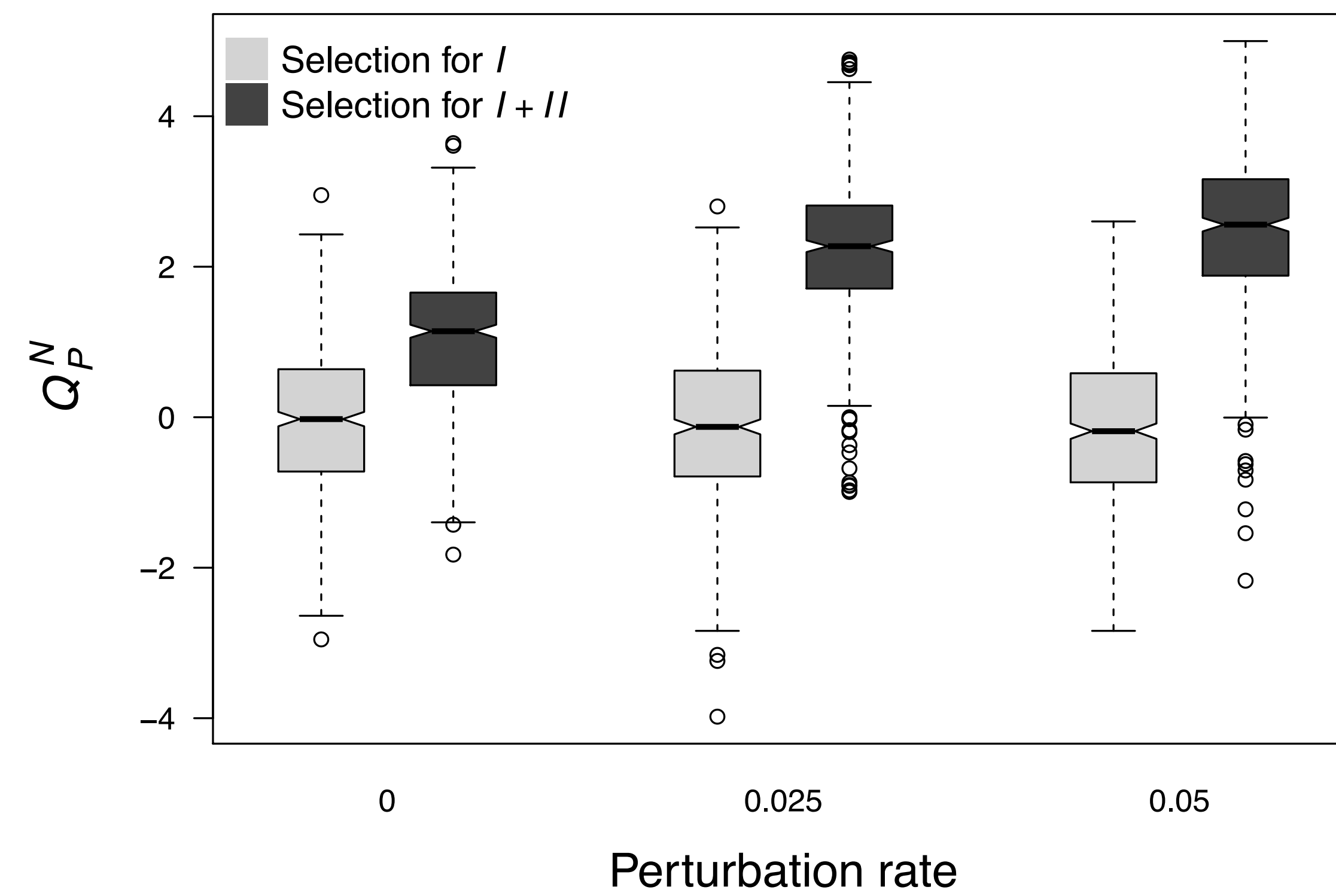

Supplement: S1 Fig — The data for this figure considers a network with the highest fitness in each population, irrespective of whether this fitness surpassed a threshold of 0.9. The results are qualitatively the same as those presented in the main text. (A) Modularity evolves after selection for an additional activity pattern. The evolutionary scenario is the same as that presented in Fig 3 in the main text (GAPs in Fig 3A). Mean ± SD QPN is lower in ancestral populations under selection for GAP I (−0.164 ± 1.009) than in populations evolved under selection for GAPs I and II (2.458 ± 0.988). Wilcoxon signed-rank test: W = 124, 950; p < 2 × 10^−16^. (B) Selection for two GAPs produces a greater increase in modularity in sparser networks. The evolutionary scenario is the same as that presented in Fig 4 in the main text (GAPs in Fig 3A). (C) The rate of perturbation of initial system states contributes to the increase in modularity due to selection for two GAPs. The evolutionary scenario is the same as that presented in Fig 5 in the main text (GAPs in Fig 3A). (PDF) [file pcbi.1006172.s002.pdf]

**A**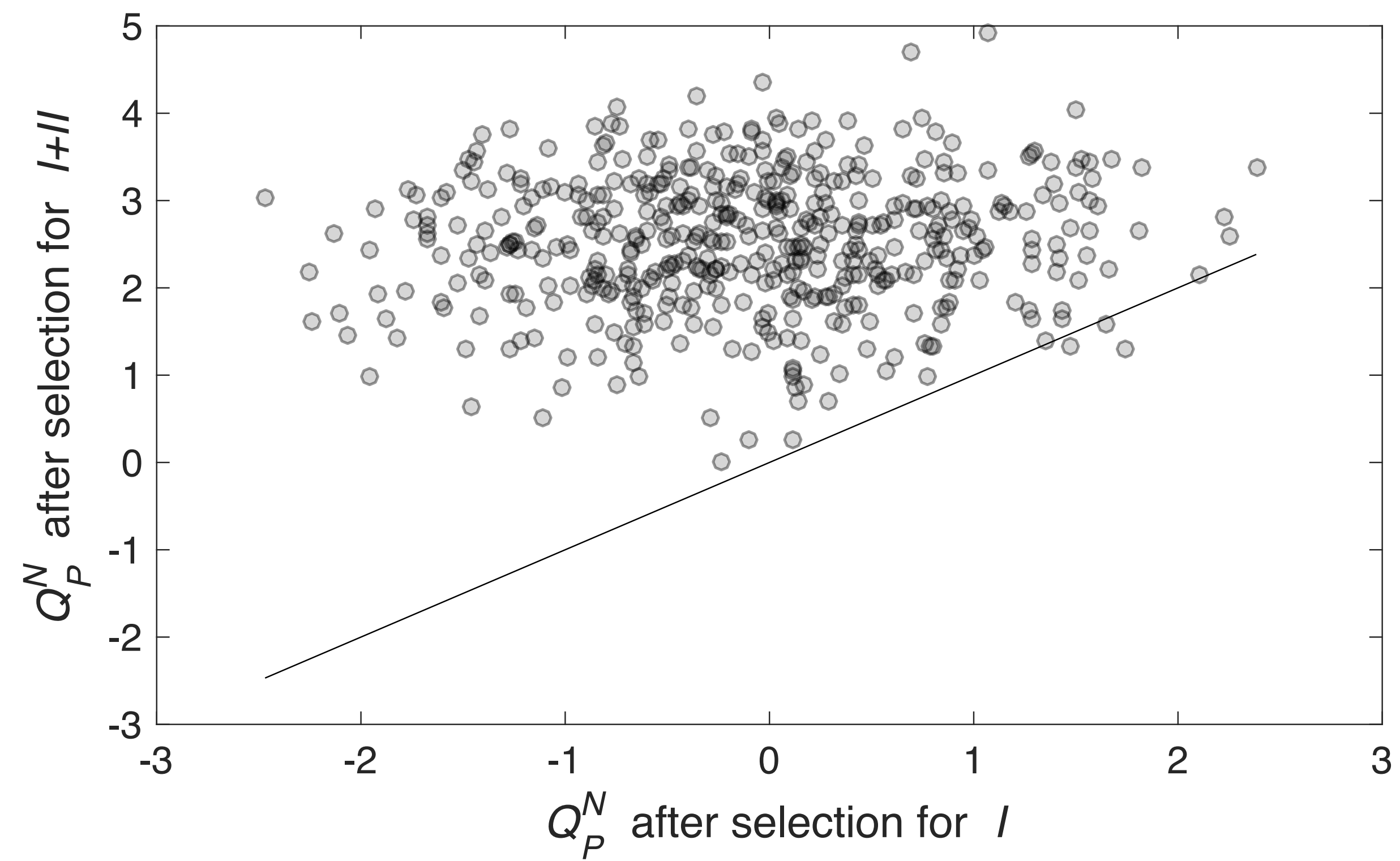**B**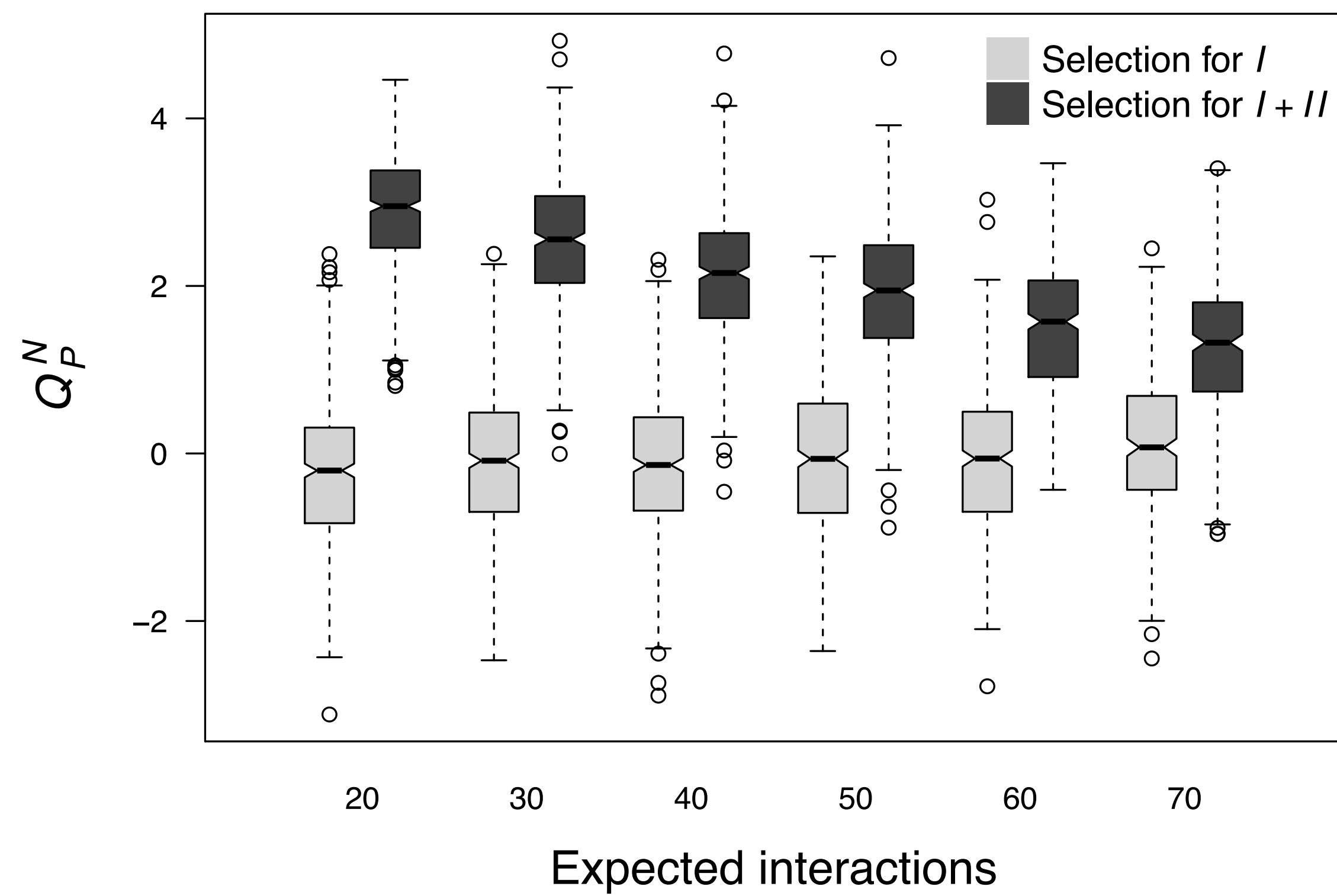**C**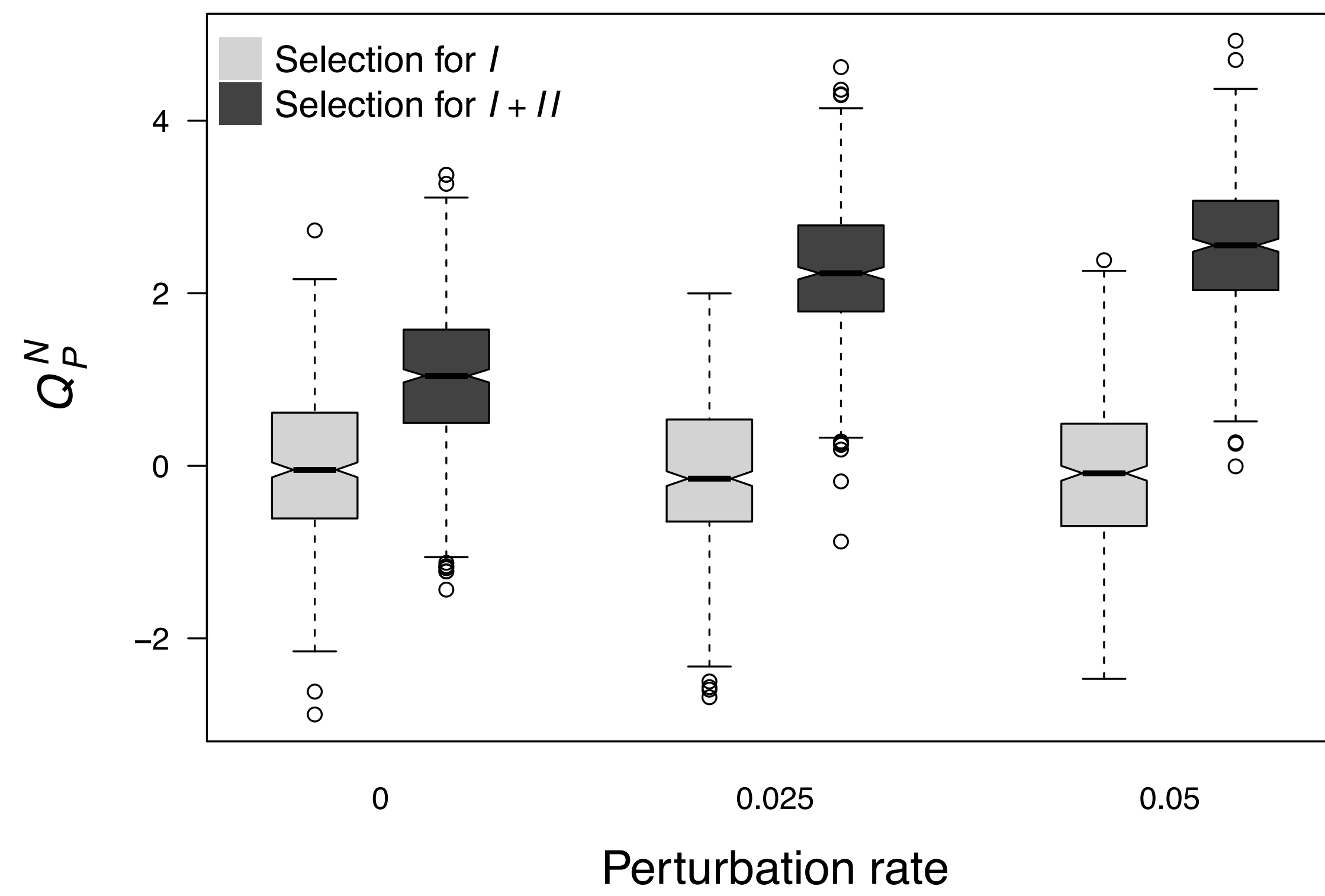

Supplement: S2 Fig — The data for this figure considers population averages in those populations where maximum fitness surpassed a threshold of 0.9. The results are qualitatively the same as those presented in the main text. (A) Modularity evolves after selection for an additional activity pattern. The evolutionary scenario is the same as that presented in Fig 3 in the main text (GAPs in Fig 3A). Mean ± SD QPN is lower in ancestral populations under selection for GAP I (−0.098 ± 0.895) than in populations evolved under selection for GAPs I and II (2.524 ± 0.773). Wilcoxon signed-rank test: W = 113, 030; p < 2 × 10^−16^. (B) Selection for two GAPs produces a greater increase in modularity in sparser networks. The evolutionary scenario is the same as that presented in Fig 4 in the main text (GAPs in Fig 3A). (C) The rate of perturbation of initial system states contributes to the increase in modularity due to selection for two GAPs. The evolutionary scenario is the same as that presented in Fig 5 in the main text (GAPs in Fig 3A). (PDF) [file pcbi.1006172.s003.pdf]

**A**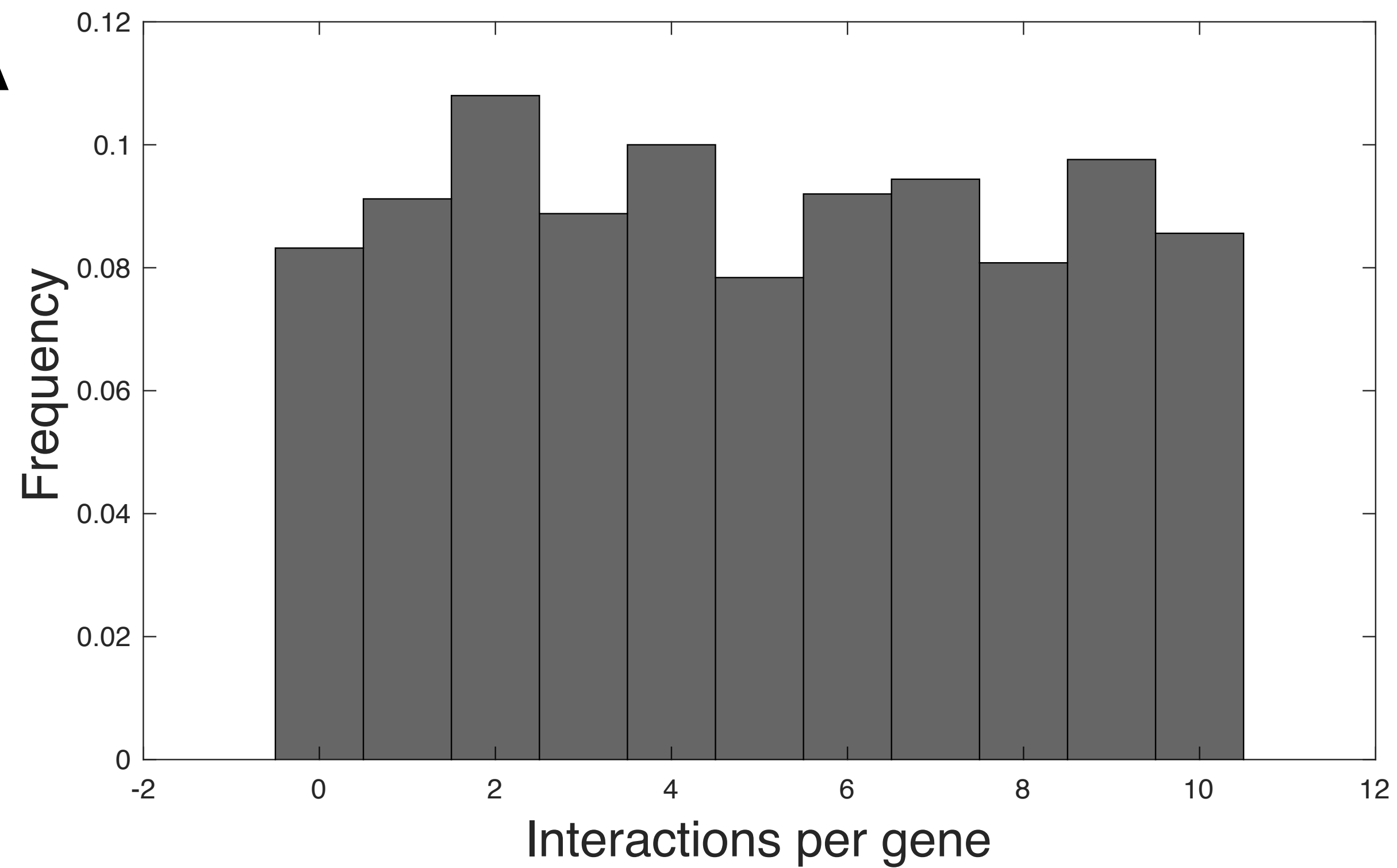**B**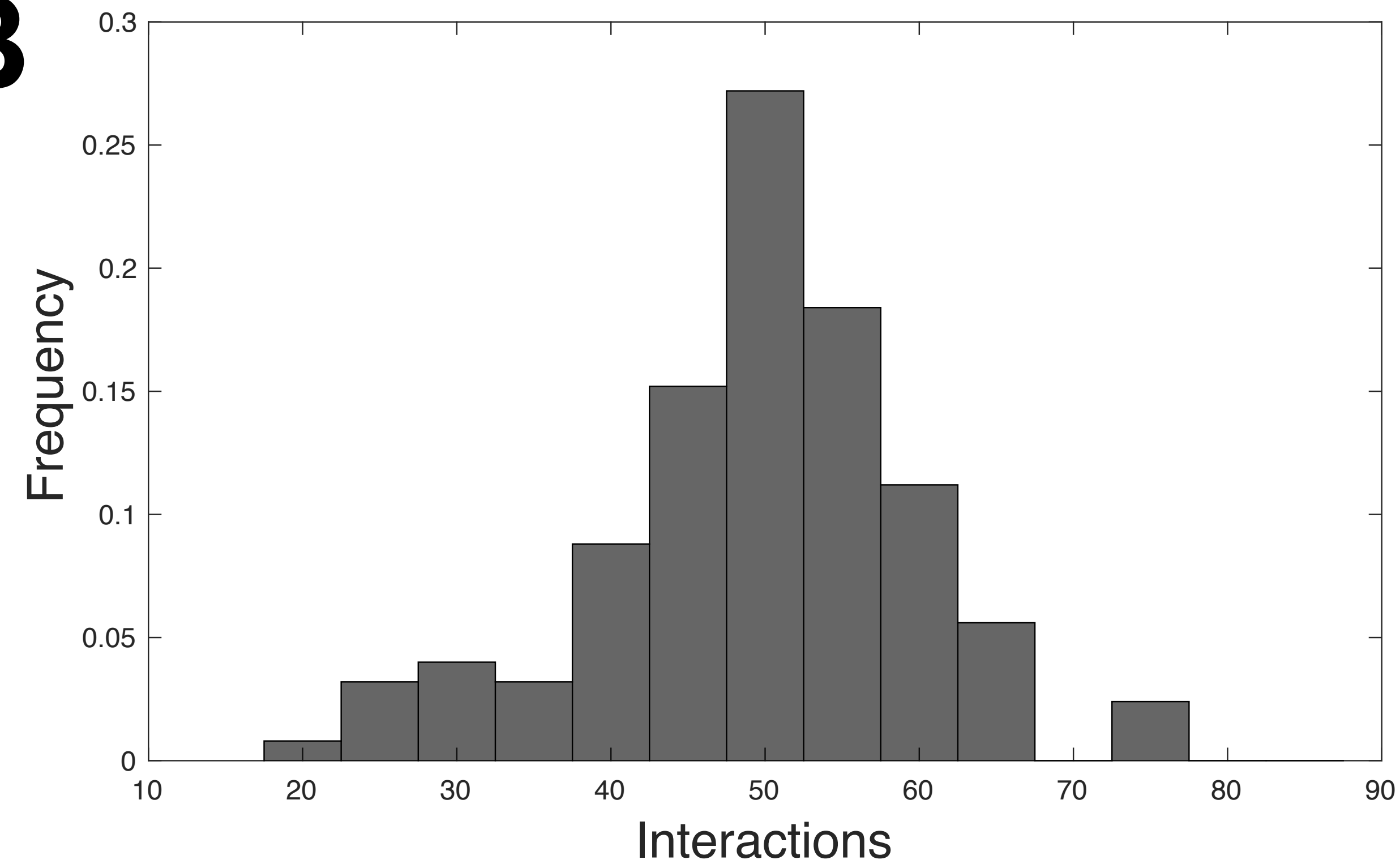**C**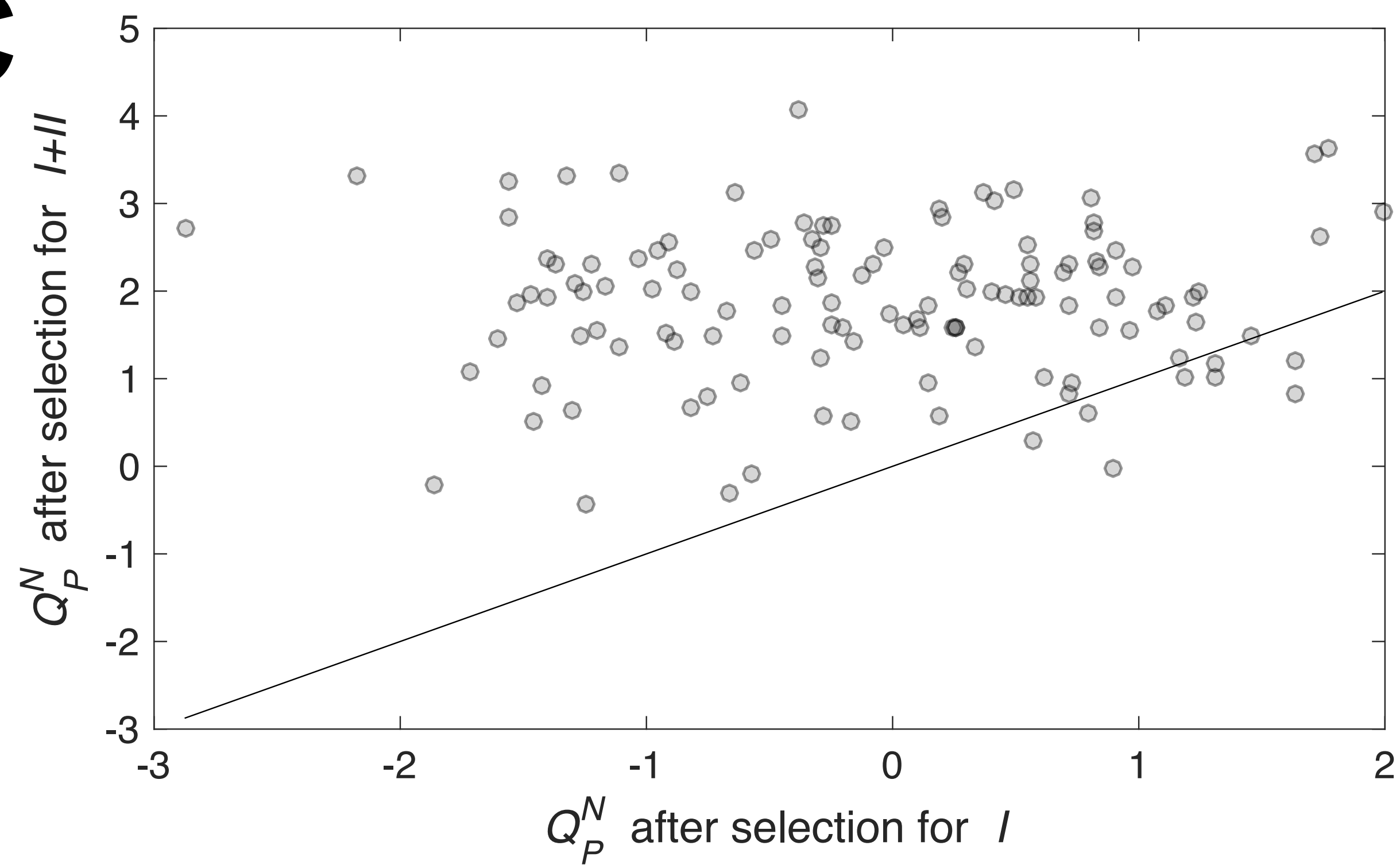**D**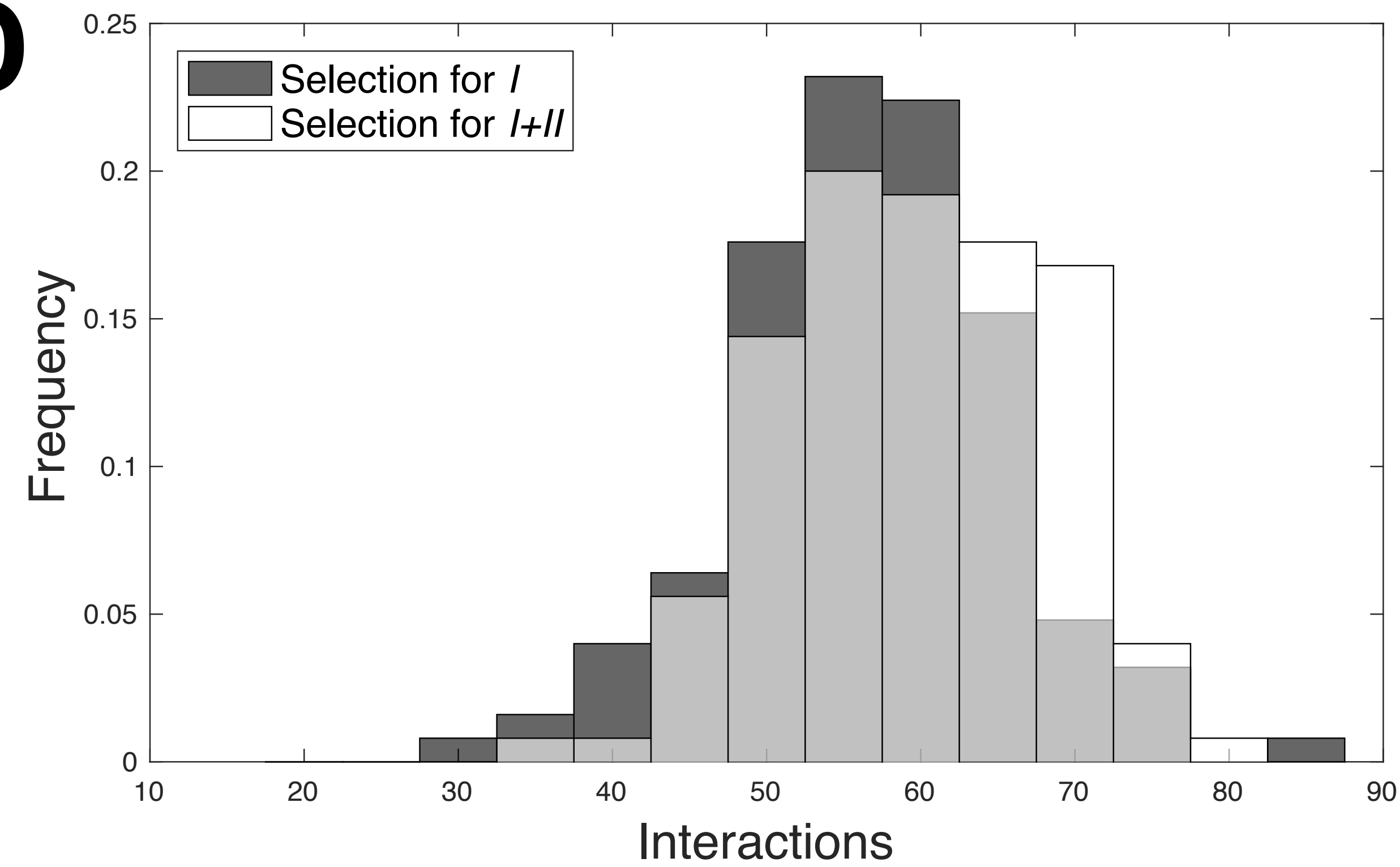

Supplement: S3 Fig — For the results in this figure, mutation was implemented in a different manner than in the rest of the paper. Here, a gene undergoing mutation acquired or lost an interaction with equal probabilities, regardless the number of regulators it has. The figure shows that, even when mutation is not biased to a particular number of regulators per gene, the number of interactions does not decrease beyond random expectation when modularity evolves after selection for an additional GAP. Results in panels A and B consider 125 populations that evolved 8,000 generations in the absence of selection. Panels C and D refer to 125 populations that evolved in a similar scenario as populations described in Fig 3 in the main text. That is, they evolved first under selection for a single GAP (GAP I in Fig 3A) and then under selection for two GAPs (I and II in Fig 3A). (A) Distribution of the number of regulators per gene after 8,000 generations of neutral evolution. The figure is coherent with a uniform distribution for the number of regulators per gene with this mutation set up. (B) Distribution of the number of interactions per network. The mean number of interactions per network is 49.592 (SD = 10.21). This expected value is thus close to the theoretical expectation of 50 interactions per network. (C) QPN increases significantly after selection for an additional activity pattern. Specifically, it increases from −0.077 ± 0.98 to 1.875 ± 0.865 (W = 7, 778; p < 2.2 × 10^−16^). (D) The number of interactions after selection for either one or two GAPs is not lower than in the absence of selection (compare to panel B). The mean ± SD number of interactions is 56.42 ± 8.88 after selection for GAP I and 59.67 ± 8.5 after selection for GAPs I and II. (PDF) [file pcbi.1006172.s004.pdf]

# A

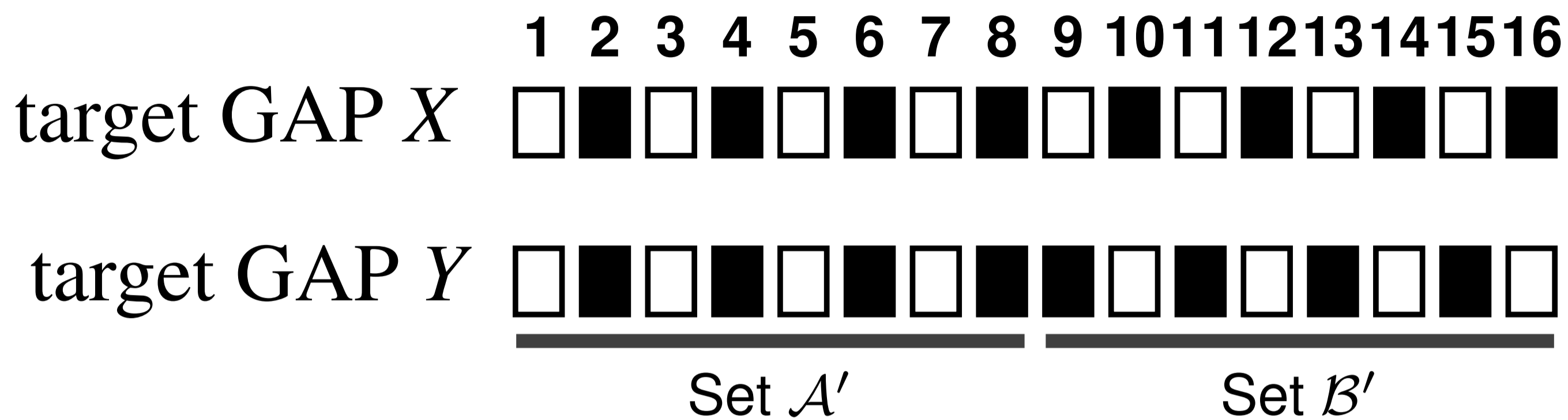

# B

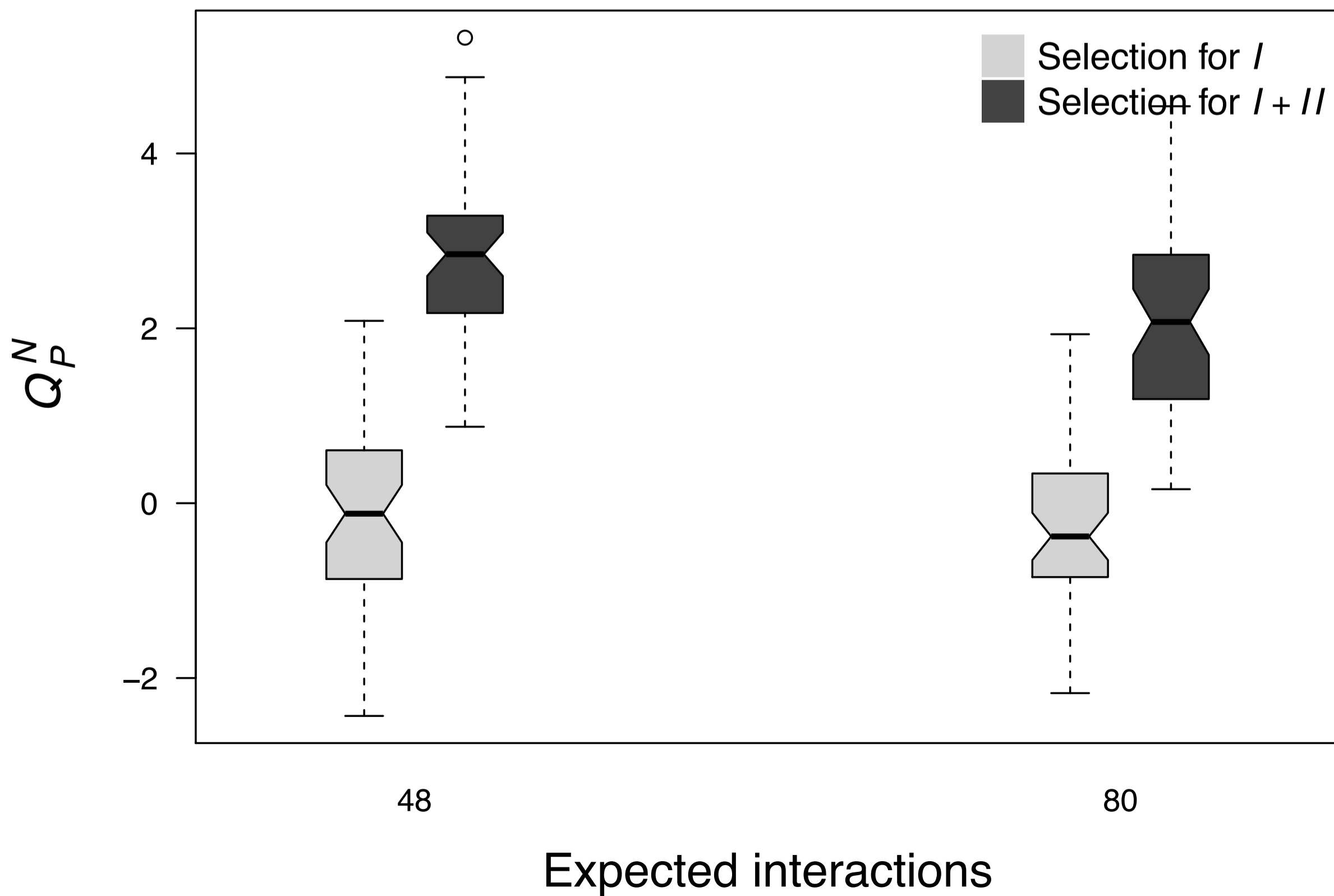

Supplement: S4 Fig — For the simulations in this figure the parameters that I used were the following: N = 16, μ = 0.02, κ = 0.03, S = 0.8, K = 50/T. (A) Target GAPs X and Y. Genes 1-8 are grouped in set A′ and genes 9-16 are grouped in set B′. Note that genes in set A′ have the same activity state in both target GAPs and genes in set B′ have a different activity state in both target GAPs. (B) Sparseness contributes to modularity after selection for an additional GAP. The figure compares results for 50 populations evolved under a propensity to gain interactions γ = 3/16 (48 expected interactions) and 50 populations evolved under a propensity to gain interactions γ = 5/16 (80 expected interactions). Network populations evolved first under selection to yield target GAP X for 1,500 generations. In a second stage that lasted 7,500 generations, selection favoured networks that produce GAPs X and Y from different initial system states. (PDF) [file pcbi.1006172.s005.pdf]

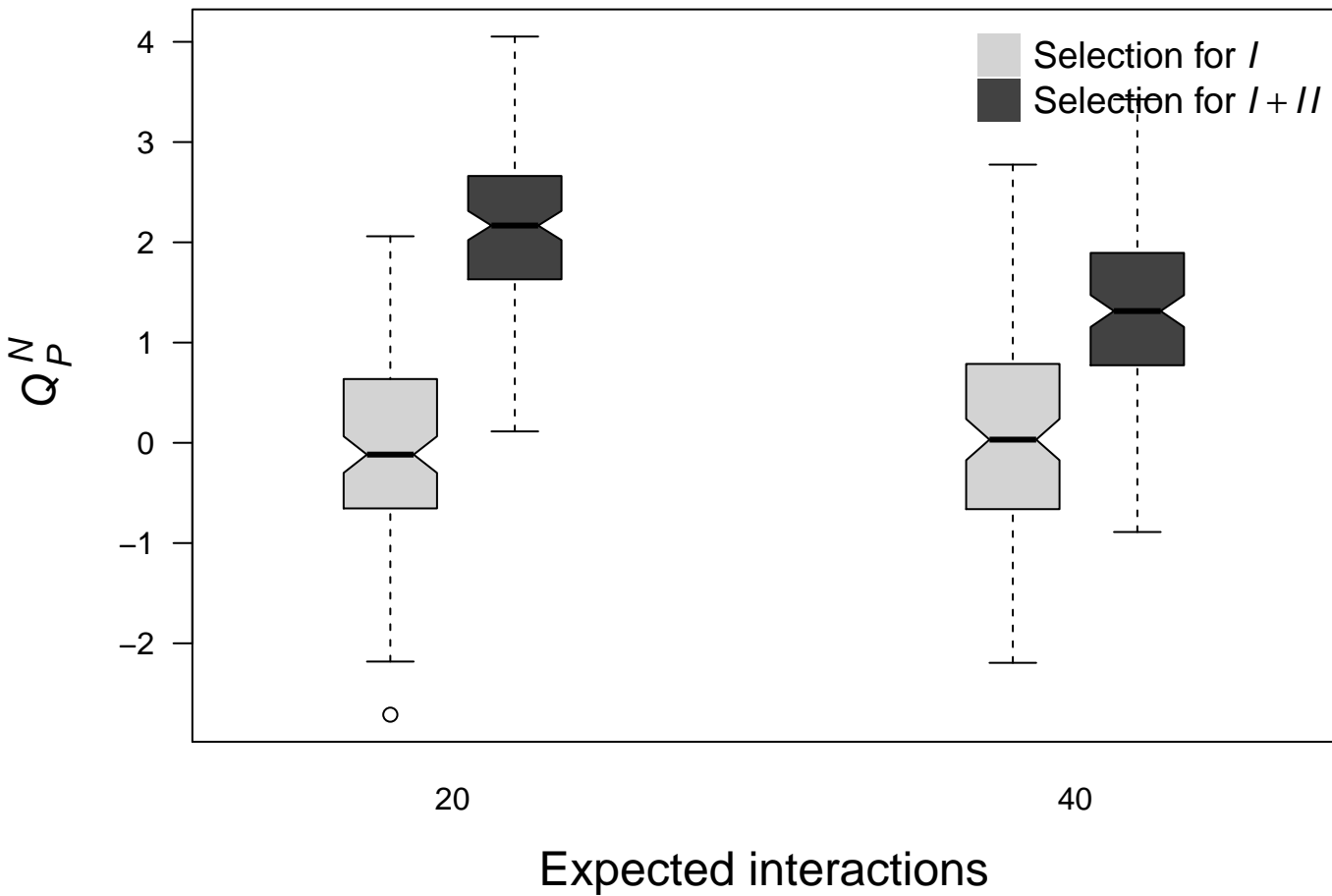

Supplement: S5 Fig — For the simulations in this figure the value of θi is set to 0 for all genes. Therefore, network dynamics is given by sit+1=σi[∑j=1Ngijsjt], where the function σi(x) equals 1 when x > 0, it equals sit when x = 0 and it equals 0 when x < 0. The figure compares results for 125 populations evolved under a propensity to gain interactions γ = 0.2 (20 expected interactions) and 125 populations evolved under a propensity to gain interactions γ = 0.4 (40 expected interactions). The evolutionary scenario is the same as that presented in Figs 3 and 4 in the main text (GAPs in Fig 3A), in which populations evolved first under selection for a single GAP (GAP I) for 2,000 generations and then under selection for two GAPs (GAPs I and II) for 8,000 generations. The figure shows that, also with this model, selection for two GAPs produces a greater increase in modularity in sparser networks. (PDF) [file pcbi.1006172.s006.pdf]

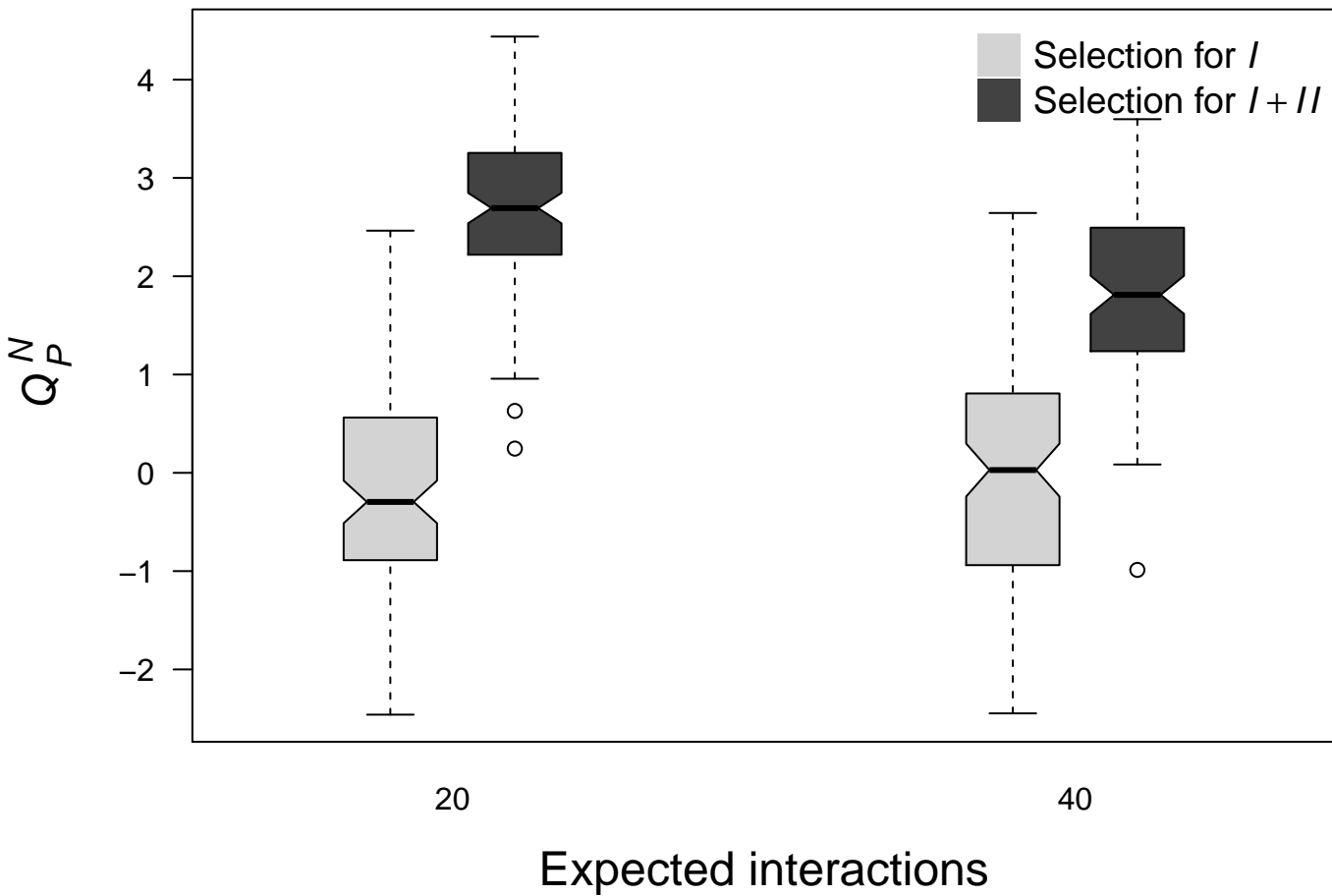

Supplement: S6 Fig — For the simulations in this figure, the probability that a regulation of gene i is lost equals μ(1-γ)RiN. The probability that an interaction is acquired is μγN-RiN. In this case, the new weight of the interaction is taken from an N(0,1) distribution. The probability that the weight of an interaction is modified by mutation is μγRiN. When an existing interaction is modified, the new weight is taken from an N(0,1) distribution, but its sign is forced to be the same that it had before mutation. The figure compares results for 125 populations evolved under a propensity to gain interactions γ = 0.2 (20 expected interactions) and 125 populations evolved under a propensity to gain interactions γ = 0.4 (40 expected interactions). The evolutionary scenario is the same as that presented in Figs 3 and 4 in the main text (GAPs in Fig 3A), in which populations evolved first under selection for a single GAP (GAP I) for 2,000 generations and then under selection for two GAPs (GAPs I and II) for 8,000 generations. The figure shows that, also with this model, selection for two GAPs produces a greater increase in modularity in sparser networks. (PDF) [file pcbi.1006172.s007.pdf]

**A**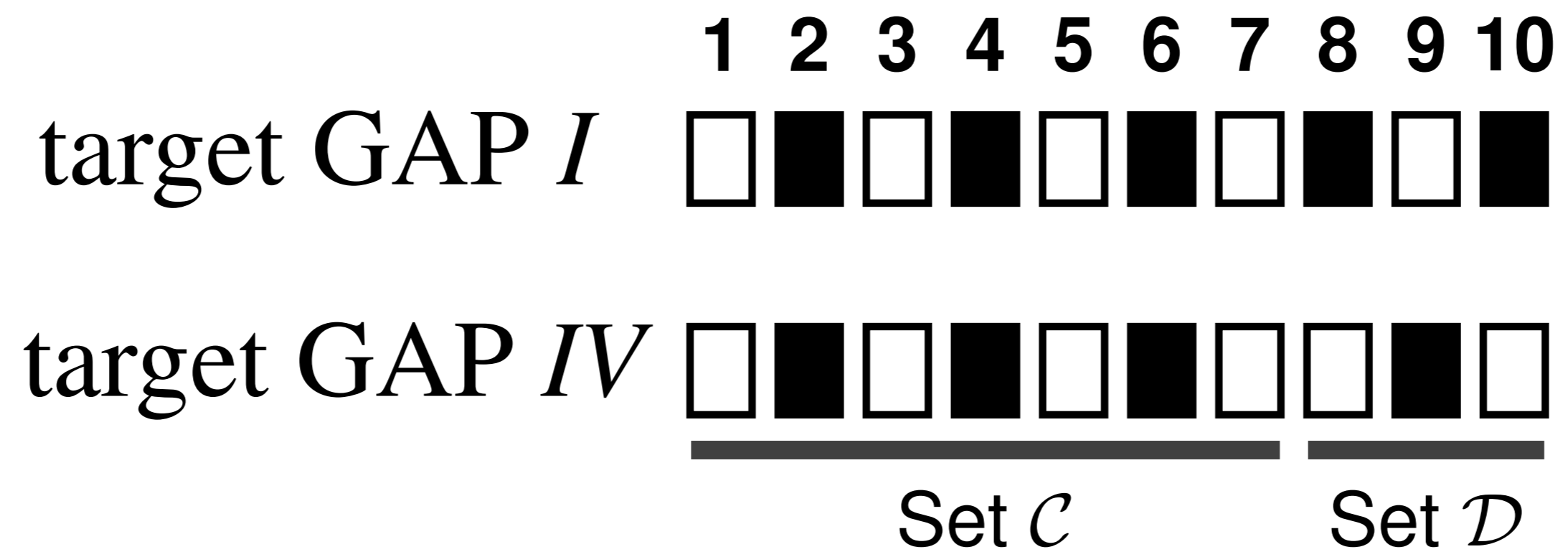**B**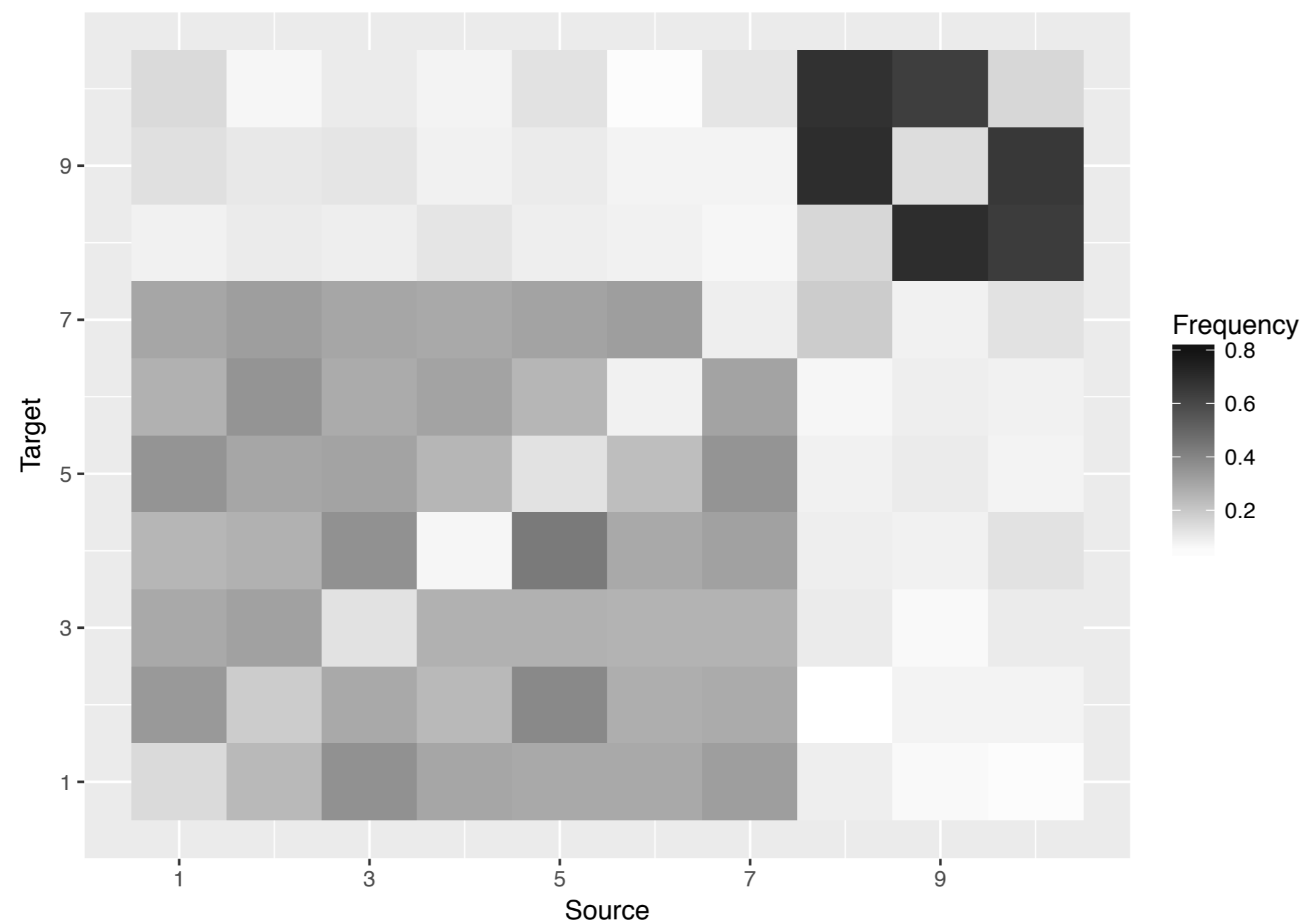**C**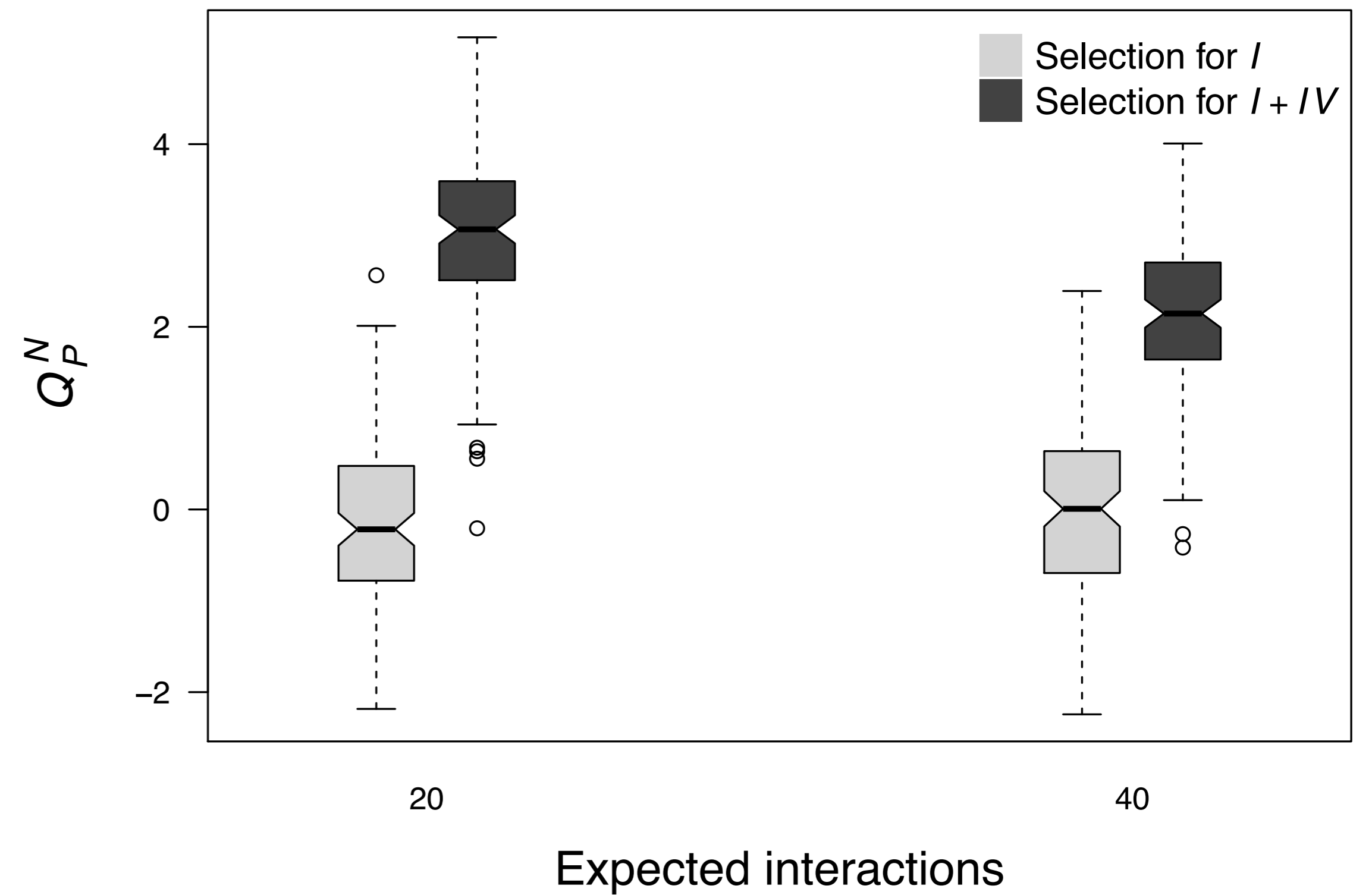

Supplement: S7 Fig — (A) Target GAPs I and IV. Genes 1-7 are grouped in set C and genes 8-10 are grouped in set D. Note that genes in set C have the same activity state in both target GAPs. In contrast, the three genes in set D have a different activity state in both target GAPs. Networks evolve in a first stage under selection to produce target GAP I. In a second stage, selection favours networks that produce target GAPs I and IV from distinct initial system states. (B) After selection for both target GAPs I and IV, interactions occur mainly either between genes in set C or between genes in set D. (C) Selection for two GAPs produces a greater increase in modularity in sparser networks. (PDF) [file pcbi.1006172.s008.pdf]

**A**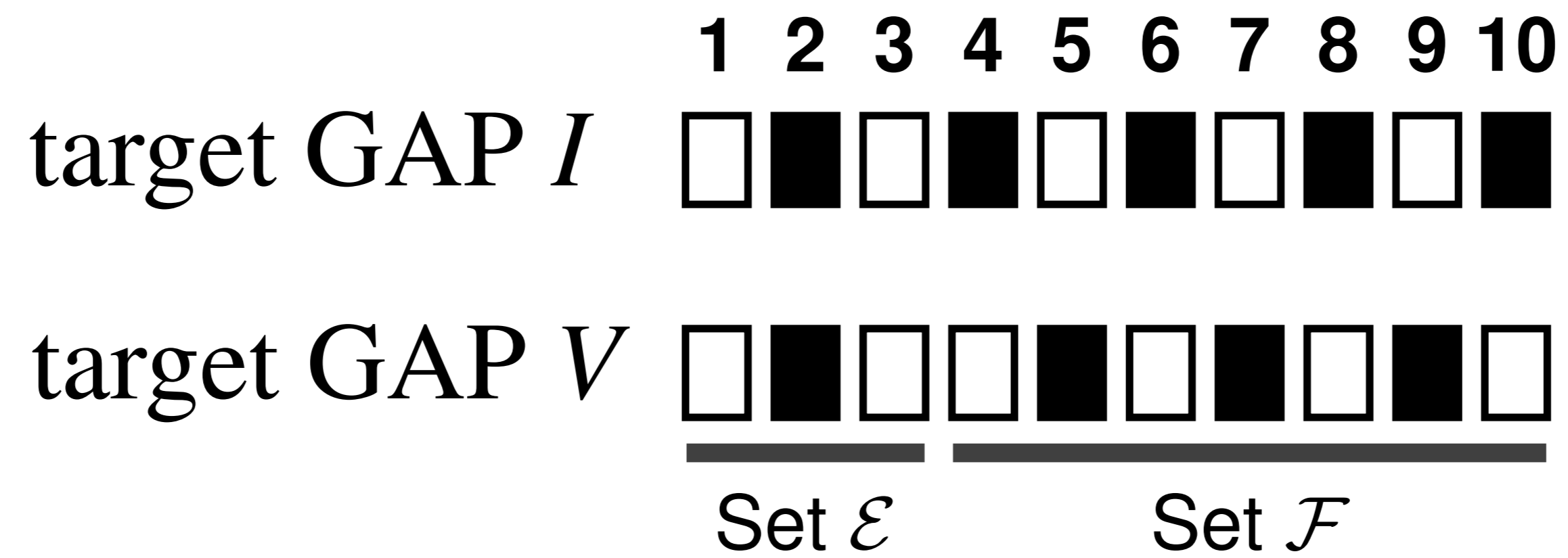**B**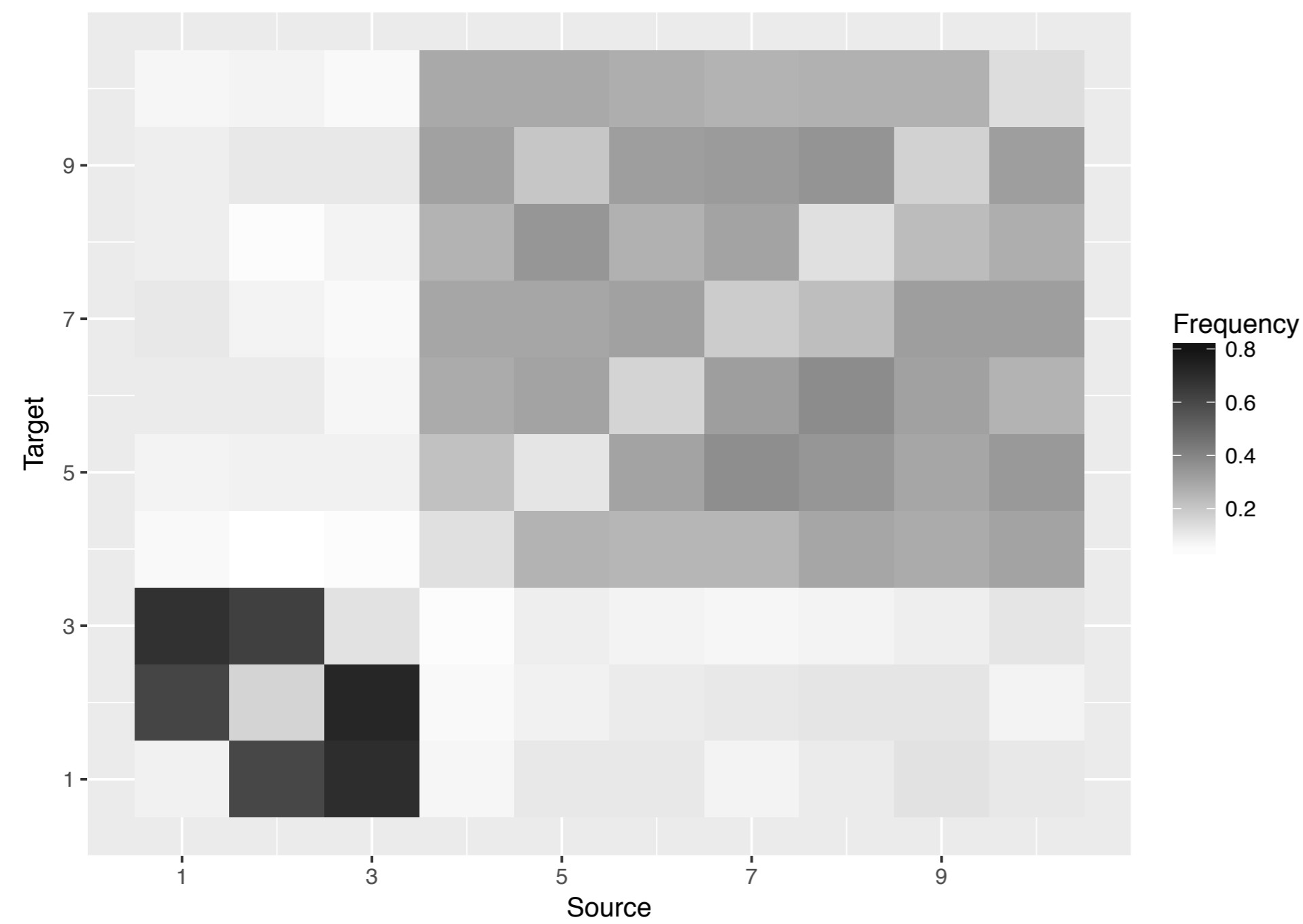**C**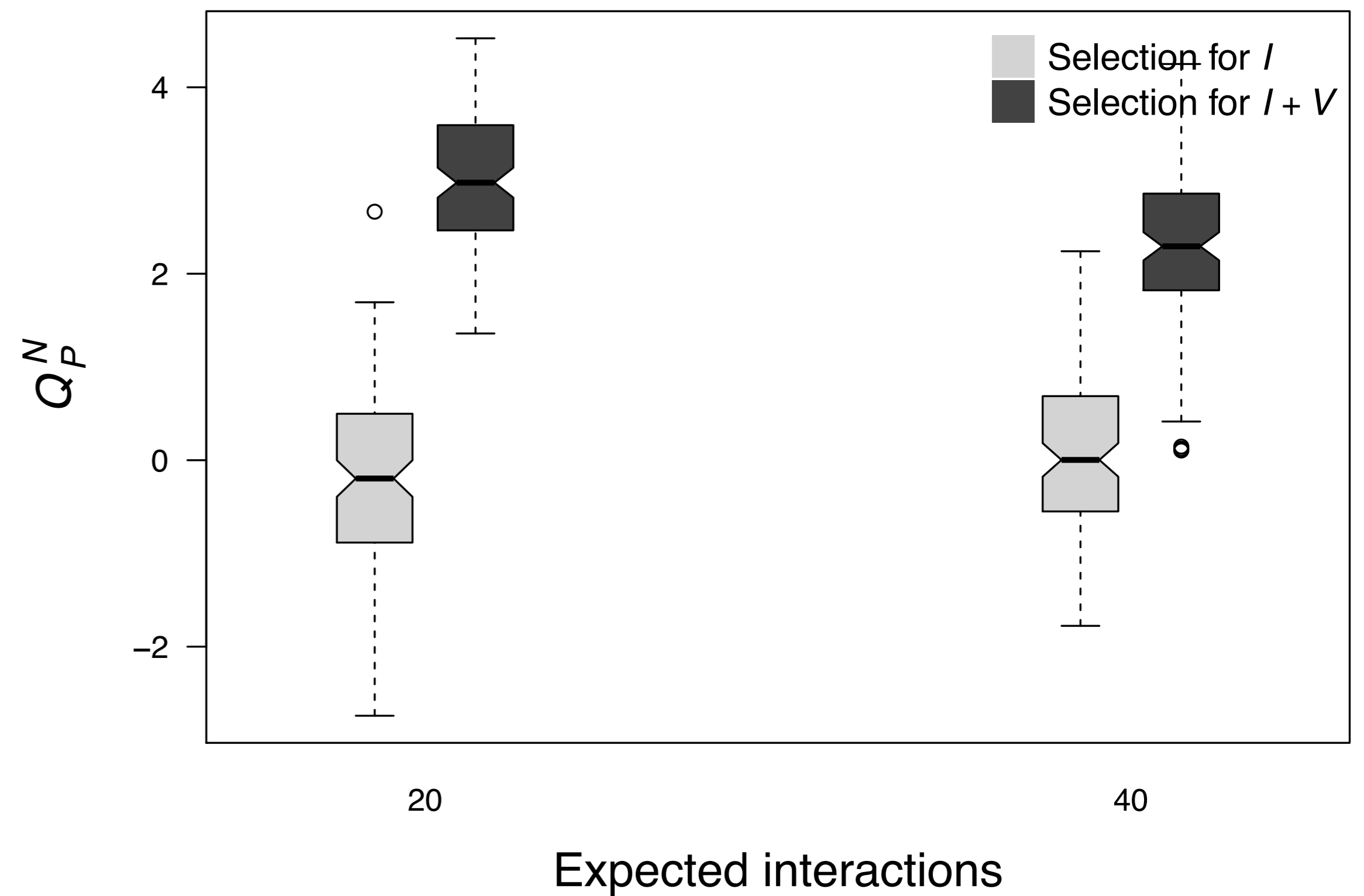

Supplement: S8 Fig — (A) Target GAPs I and V. Genes 1-3 are grouped in set E and genes 4-10 are grouped in set F. Note that genes in set E have the same activity state in both target GAPs. In contrast, the seven genes in set F have a different activity state in both target GAPs. Networks evolve in a first stage under selection to produce target GAP I. In a second stage, selection favours networks that produce target GAPs I and V from distinct initial system states. (B) After selection for both target GAPs I and V, interactions occur mainly either between genes in set E or between genes in set F. (C) Selection for two GAPs produces a greater increase in modularity in sparser networks. (PDF) [file pcbi.1006172.s009.pdf]
